# Estimation of flint hills tallgrass prairie productivity and fuel loads: a model-based synthesis and extrapolation of experimental data

**DOI:** 10.1007/s10980-024-02034-4

**Published:** 2025-01-15

**Authors:** Robert B. McKane, Jonathan J. Halama, Bradley L. Barnhart, Allen F. Brookes, Kevin S. Djang, Sonali Chokshi, Paul P. Pettus, Brenda Groskinsky, Gina Grier, Andy Hawkins, Douglas Watson, Jayson Prentice, John M. Blair, Douglas G. Goodin, Loretta C. Johnson, Adam M. Skibbe, Marc Stieglitz, Feifei Pan, Alex Abdelnour

**Affiliations:** U.S. Environmental Protection Agency, Office of Research and Development, Corvallis, OR 97333, USA; U.S. Environmental Protection Agency, Office of Research and Development, Corvallis, OR 97333, USA; U.S. Environmental Protection Agency, Office of Research and Development, Corvallis, OR 97333, USA; U.S. Environmental Protection Agency, Office of Research and Development, Corvallis, OR 97333, USA; Inoventures, Inc., Corvallis, OR 97333, USA; U.S. Environmental Protection Agency, Office of Research and Development, Corvallis, OR 97333, USA; U.S. Environmental Protection Agency, Office of Research and Development, Corvallis, OR 97333, USA; U.S. Environmental Protection Agency Region 7, Lenexa, KS 66219, USA; U.S. Environmental Protection Agency Region 7, Lenexa, KS 66219, USA; U.S. Environmental Protection Agency Region 7, Lenexa, KS 66219, USA; Kansas Department of Health and Environment, Topeka, KS 66612, USA; Kansas Department of Health and Environment, Topeka, KS 66612, USA; Kansas State University, Manhattan, KS 66506, USA; Kansas State University, Manhattan, KS 66506, USA; Kansas State University, Manhattan, KS 66506, USA; Kansas State University, Manhattan, KS 66506, USA; Georgia Institute of Technology, Atlanta, GA 30332, USA; Georgia Institute of Technology, Atlanta, GA 30332, USA; University of North Texas, Denton, TX, USA

**Keywords:** Tallgrass prairie, VELMA, Konza prairie, Flint hills, NPP, Fuel loads, Air quality, Nitrogen, Soil moisture, Grazing

## Abstract

**Context:**

The > 25,000 km^2^ Flint Hills ecoregion in eastern Kansas and northeastern Oklahoma, USA, is an economically and ecologically important area encompassing the largest remaining tallgrass prairie ecosystem in North America. Prescribed fires are used routinely to control invasive woody species and improve forage production for the beef-cattle industry. However, burning releases harmful pollutants that, at times, contribute to air quality problems for communities across a multi-state area.

**Objectives:**

Establish a modeling framework for synthesizing long-term ecological data in support of Flint Hills tallgrass prairie management goals for identifying how much, where, and when rangeland burning can be conducted to maximize ecological and economic benefits while minimizing regional air quality impacts.

**Methods:**

We used EPA’s VELMA ecohydrology model to synthesize long-term experimental data at the 35 km^2^ Konza Prairie Biological Station (KPBS) describing the effects of climate, fire, grazing, topography, and soil moisture and nutrient dynamics on tallgrass prairie productivity and fuel loads; and to spatially extrapolate that synthesis to estimate grassland productivity and fuel loads across the nearly 1000 times larger Flint Hills ecoregion to support prescribed burning smoke trajectory modeling using the State of Kansas implementation of the U.S. Forest Service BlueSky framework.

**Results:**

VELMA provided a performance-tested synthesis of KPBS data from field observations and experiments, thereby establishing a tool for regionally simulating the combined effects of climate, fire, grazing, topography, soil moisture, and nutrients on tallgrass prairie productivity and fuel loads. VELMA’s extrapolation of that synthesis allowed difficult-to-quantify fuel loads to be mapped across the Flint Hills to support environmental decision making, such as forecasting when, where, and how prescribed burning will have the least impact on downwind population centers.

**Conclusions:**

Our regional spatial and temporal extrapolation of VELMA’s KPBS data synthesis posits that the effects of integrated ecohydrological processes operate similarly across tallgrass prairie spatial scales. Based on multi-scale performance tests of the VELMA-BlueSky toolset, our multi-institution team is confident that it can assist stakeholders and decision makers in realistically exploring tallgrass prairie management options for balancing air quality, tallgrass prairie sustainability, and associated economic benefits for the Flint Hills ecoregion and downwind communities.

## Introduction

Grasslands of various types are globally extensive and found on every continent except Antarctica. They generally feature a large diversity of flora and fauna in their native state, and provide food and other vital resources for human use under various intensities of agricultural management. The Flint Hills tallgrass prairie ecosystem in eastern Kansas and northeastern Oklahoma, U.S., is an economically and ecologically important area in the Central Great Plains, encompassing the largest (25,730 km^2^) remaining contiguous remnant of the once vast North American tallgrass prairie ecosystem (4% of 688,000 km^2^) that existed before European settlement (Knapp et al. (1998). Being too rocky to plow for crop production, the dominant land-use is cattle crazing. It is a prime example of a highly productive native grassland managed for grazing and livestock production.

Historically, frequent wildfires and burning by Indigenous people were essential to the development and maintenance of the North American tallgrass prairie ecosystem. Prescribed prairie fires are used routinely today in the Flint Hills to control invasive woody species and improve forage production for the multi-billion dollar beef-cattle industry. Prescribed burning is also critical for maintaining habitat for grassland birds, pollinators, and other conservation goals.

However, grassland fires can release harmful pollutants such as volatile organic compounds, nitrogen oxides (resulting in production of ozone), and particulate matter into the atmosphere, at times contributing to air quality problems for communities across a multi-state area. Consequently, the Flint Hills region has multiple stakeholder groups seeking solutions concerning when, where, and how to burn. Balancing the ecological, economic, and human health effects of rangeland burning has proven to be a major sociological and regulatory challenge. Grassland managers globally face similar challenges (e.g., Gelbard 2003).

To help address these multi-stakeholder trade-offs, the U.S. Environmental Protection Agency (EPA) Office of Research and Development (ORD) and EPA Region 7 (R7) have collaborated with the Kansas Department of Health and Environment (KDHE) and Kansas State University (KSU) to develop a model-based synthesis of tallgrass prairie experimental data for estimating Flint Hills grassland productivity and fuel loads. These data can be used as inputs for smoke dispersion and trajectory models, such as BlueSky (Larkin et al. 2009), to produce estimates of downwind air quality impacts resulting from real and hypothetical land management actions such as prescribed burning location and frequency.

One such data synthesis tool, EPA’s Visualizing Ecosystem Land Management Assessments (VELMA), is an ecohydrological model linking hydrologic and biogeochemical processes in watersheds. It is applicable to essentially any terrestrial ecosystem—e.g., grasslands, forests, urban, wetland, tundra, mixed-use watersheds—for which sufficient calibration data are available to accurately simulate plant-soil–water interactions and responses to daily and interannual changes in land use and climate (e.g., Abdelnour et al. 2011, 2013; McKane et al. 2014; 2018a, b, 2020, 2022; Barnhart et al. 2015, 2021; Yee et al. 2017; Hoghooghi et al. 2018; U.S. EPA 2021; Oregon Department of Fish and Wildlife 2022; Halama et al. 2023, 2024).

For the Flint Hills ecoregion, we calibrated VELMA for tallgrass prairie based on over four decades of published data and supporting databases for the Konza Prairie Biological Station (KPBS), a 35 km^2^ protected area of native tallgrass prairie located within the Flint Hills of northeastern Kansas and part of the National Science Foundation’s Long Term Ecological Research (LTER) network since 1980 (http://lter.konza.ksu.edu/konza-prairie-long-term-ecological-research-lter).

Here we report on several tallgrass prairie VELMA modeling objectives: (1) Develop a model-based synthesis of long-term KPBS experimental data describing the effects of climate, fire, grazing, topography, and soil moisture on tallgrass prairie productivity and fuel loads; (2) Extrapolate that KPBS synthesis to estimate and couple VELMA Flint Hills high-resolution fuel loads with State of Kansas BlueSky modeling to improve existing real-time, downwind air quality impacts of prescribed burning on rural and urban communities.

Additionally, as a process-based ecosystem simulator VELMA also supports State of Kansas smoke management plans to assist rural and urban Flint Hills stakeholders and jurisdictional decision makers in their shared goals to better understand, estimate, and balance ecological and air quality trade-offs for actual versus hypothetical rangeland burning scenarios (State of Kansas 2010). In general, well calibrated process-based models are better suited for such extrapolations than statistically based fuel load models—e.g., LANDFIRE (Rollins 2009)—to the extent that such forward looking scenarios lie beyond the range of existing climatic and other environmental conditions for which statistical models have been developed (Cuddington et al. 2013).

## Methods

### Study areas

#### Konza prairie biological station (KPBS)

Applying VELMA for modeling fuel loads across the Flint Hills ecoregion began with its calibration using published data from long-term field research at KPBS. As described by Briggs and Knapp (1995), KPBS is a tallgrass prairie located in the Flint Hills region of northeast Kansas, USA, about 10 km south of Manhattan. KPBS has many features representative of the broader Flint Hills ecoregion, including a dissected landscape with steep-sided hills characterized by exposed limestone and shale strata that prevented extensive cultivation. At KPBS ridges up to 444 m in elevation are generally flat with shallow, rocky soils typified by Florence silty clay loams. The wider valleys, generally about 120 m below the ridges, have deep well-drained soils typified by Tully silty clay loams. KPBS climate is temperate midcontinental with 75% of the annual precipitation (35-yr mean = 835 mm) occurring during the April to September growing season. Vegetation is largely native tallgrass prairie dominated by big bluestem (*Andropogon gerardii*), little bluestem (*Schizachyrium scoparium*), Indian grass (*Sorghastrum nutans*), and switch grass (*Panicum virgatum*) (Freeman and Hulbert 1985).

European settlement brought major change in the tallgrass prairie grazing regime, shifting almost entirely from bison to cattle, leading to attendant changes in grassland biodiversity (Knapp et al. 1999). Pre-versus post-settlement changes in fire frequency are more difficult to estimate, but tree ring data near tallgrass prairie-forest boundaries in Oklahoma suggest that mean fire intervals between 1759 and 2003 decreased from 3.8 years in the 1700s to 2.1 years in modern times (Allen and Palmer 2011). Before KPBS was established as a research site in the 1970s, prescribed fire intervals in the area varied between 1 and 3 years (Hulbert 1988). Under a KPBS experimental plan initiated in 1971, different watersheds were assigned a variety of prescribed fire and grazing treatments encompassing modern Flint Hills rangeland management practices, including fire extremes (i.e., annual burning to long-term fire suppression) as well as cattle vs. bison grazing treatments. Additional details and references for these KPBS fire and grazing treatments are described later in this Methods section.

#### Flint hills ecoregion

**Fig. 1:**
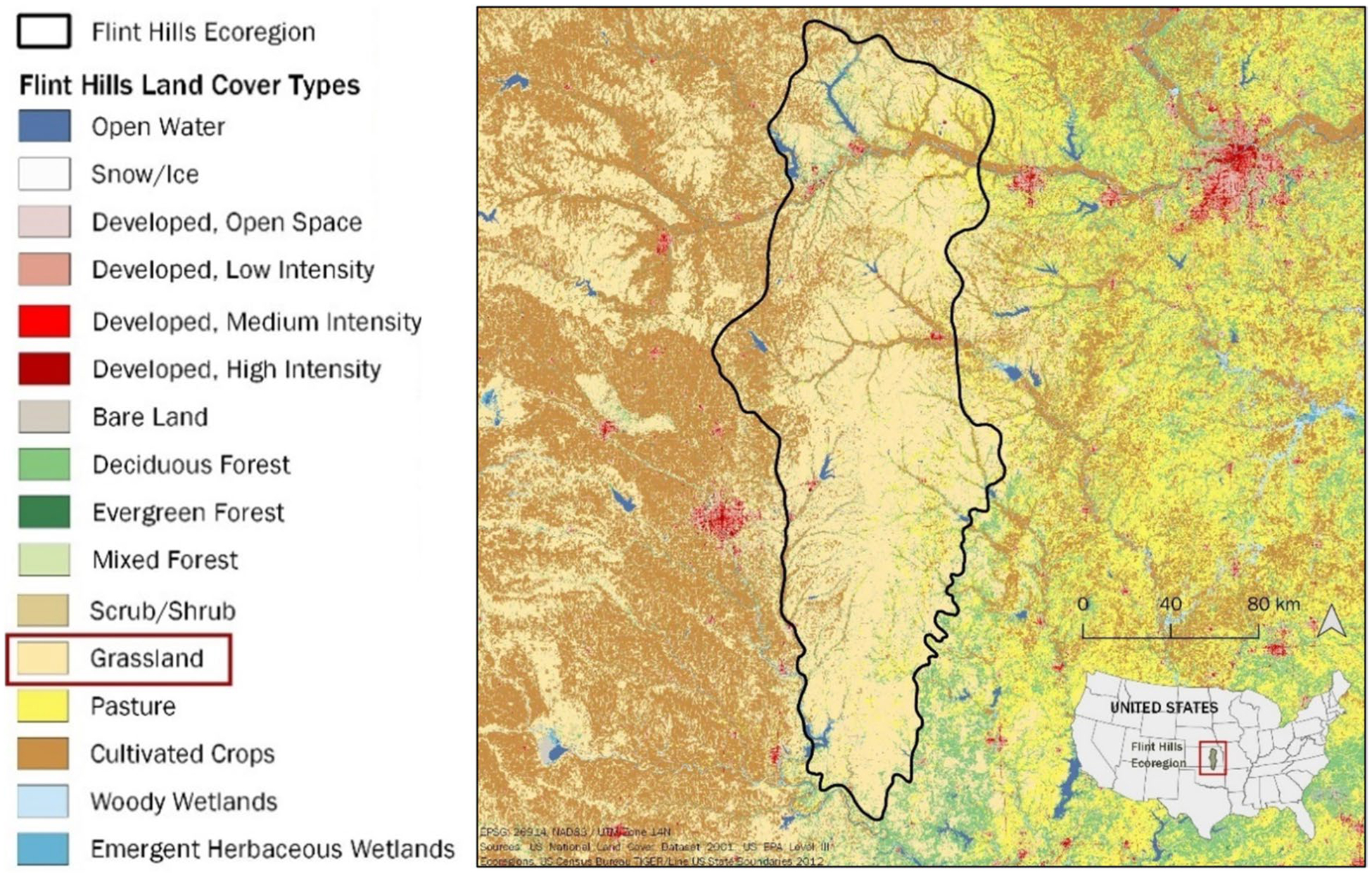
The Flint Hills ecoregion covers about 25,730 km^2^ in Kansas and Oklahoma. Source: U.S. National Land Cover Dataset 2001, public domain. NLCD classification of grassland and pasture cover types can be functionally synonymous, insofar as pasture can be subjected to periodic rangeland fires, intentionally or otherwise. As such, they are both modeled herein as fire-managed tallgrass prairie ecosystems per annual MODIS burn-scars, as delineated in Fig. 4. For example, a spatial comparison of Fig. 1 and Fig. 4 shows that most 2011 and 2014 fires occurring in Nowata County (southeast of Fig. 1 Flint Hills boundary) were primarily pasture-classified grassland

The Flint Hills ecoregion (Fig. 1) contains the largest remaining contiguous tract of tallgrass prairie, an ecosystem that once covered 170 million acres across the center of North America (Kollmorgen and Simonett 1965). Within the Flint Hills, grasslands account for 65% of the total land area, cropland 24%, woodlands 7%, with urban and other cover types accounting for 4%. In modern times, prescribed fires have mostly replaced the role of wildfire in maintaining the tallgrass prairie (Briggs and Knapp 1995); and cattle have mostly replaced bison as the dominant grazer, though not the keystone role bison played in tallgrass prairie ecology (Knapp et al. 1999; Ratajczak et al. 2022).

Nonetheless, the importance of periodic fires for maintaining tallgrass prairie is unquestioned. Without fire, eastern redcedar (*Juniperus virginiana L*.) and other woody species (*Cornus drummundii*) rapidly establish and convert prairie to shrublands or closed canopy forest stands (Ratajczak et al. 2014) that are extremely difficult to restore to tallgrass prairie (Collins et al. 2021). Tallgrass prairie rangeland management is well-adapted for maintaining sustainable rangelands for forage production, as described below under the Methods section, *VELMA Set Up for Simulating Flint Hills ANPP and Fuel Loads*.

## Model

### VELMA description

**Fig. 2:**
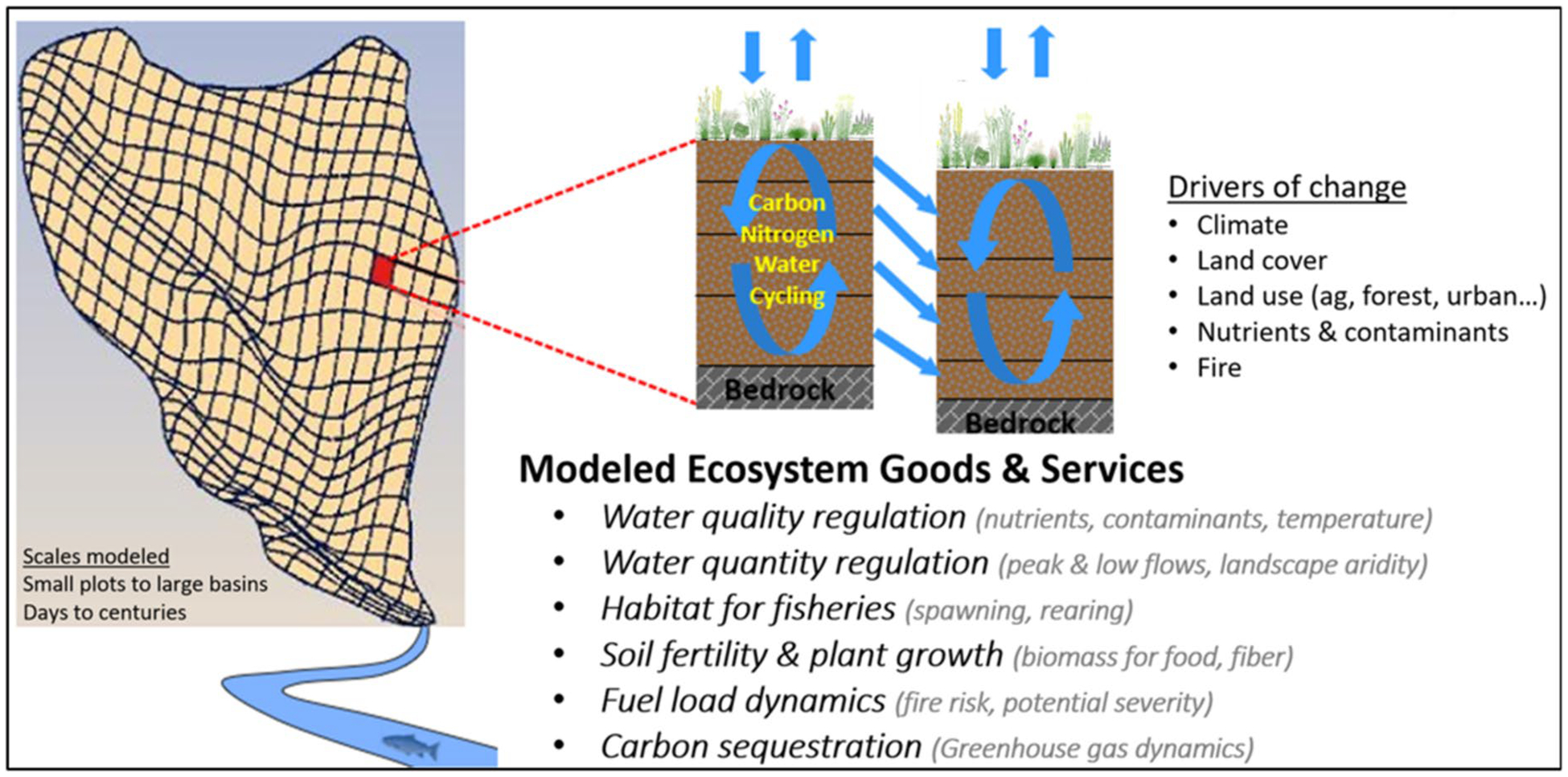
VELMA conceptual modeling framework integrating plant-soil–water ecohydrological processes for simulating effects of climate, land cover, and management on soil moisture, C and N cycling, plant productivity, and ecosystem goods and services. Each VELMA grid cell (30-m and 125-m for KPBS and Flint Hills, respectively) has 4 soil layers having specified physical, chemical, and hydrologic parameters; and specified vegetation cover type and biomass properties (McKane et al. 2014)

The VELMA model (Fig. 2) is a spatially distributed (grid-based) ecohydrological model that simulates integrated daily responses of vegetation, soil, and hydrologic components to changes in climate, land use, and land cover by linking a land surface hydrology model with a terrestrial biogeochemistry model (McKane et al. 2014). The near surface (< 5 m) hydrology model simulates water infiltration and redistribution within 4 soil layers, evapotranspiration (ET), and surface and subsurface runoff to streams. The biogeochemistry model simulates plant growth and mortality, formation and turnover of detritus and soil organic matter, and associated cycling of nitrogen (N) and carbon (C). VELMA simulates the effects of management activities on cycling and transport of N, C, and water within plots, hillslopes, and watersheds, with consequent topographic effects on plant, soil, and soil moisture dynamics. The integration of hydrological and biogeochemical processes in the model constrains changes in ecosystem structure and function in response to environmental changes. For the KPBS and Flint Hills studies, modeled land-management scenarios included prescribed fire and wildfire, cattle and bison grazing of grassland forage, and associated manure and urine N deposition and grazer weight gain. VELMA simulates these land management activities in a spatially and temporally explicit manner, incorporating defined boundaries and timing of natural and managed ecosystem disturbances (McKane et al. 2016). VELMA has been applied in many terrestrial ecosystem types, including grasslands, agricultural lands, forests, floodplains, alpine, urban, and watersheds supporting mixed land uses and land covers (cited previously). These studies demonstrate how VELMA’s design and broad applicability have made it useful for simulating effects of alternative management decisions on ecosystem service trade-offs. That is, the extent to which watersheds can sustainably support multiple ecosystem services vital to human health and well-being, e.g., clean water for drinking, recreation, and fish habitat; healthy air quality; flood prevention; production of food and fiber; and reduction of greenhouse gases (Yee et al. 2017; McKane et al. 2018a, b; U.S. EPA 2021).

### VELMA Calibration Using KPBS Data

We used long-term experimental and observational data collected at the KPBS site to calibrate VELMA version 2.0.8.0 for simulating effects of climate, prescribed fire, topographic position, and cattle grazing on aboveground net primary production (ANPP) and fuel loads in a tallgrass prairie ecosystem. The integration of hydrological and biogeochemical processes in VELMA requires a top-down approach to model calibration (McKane et al. 1997). That is, the adjustment of model parameters controlling carbon (C), nitrogen (N), and water dynamics within plants and soils, such that observed plot-scale responses to change are consistent with observed ecosystem-scale responses to disturbances.

As an example, we calibrated VELMA to simultaneously simulate KPBS data describing daily and long-term changes in daily stream discharge for nested watersheds (Costigan et al. 2015), and changes in soil moisture and plant and soil C and N dynamics in response to climate, topographic position, and fire (e.g., Briggs and Knapp 1995). This calibration process involved (1) initializing VELMA tallgrass prairie plant and soil C and N pools (g/m^2^) contained in leaves, aboveground stems, belowground stems, and fine roots for upland and lowland topographic positions; (2) calibration of parameters controlling daily and annual C and N pool-to-pool transfers (fluxes) associated with growth, mortality, soil organic matter formation and decomposition, and cycling of dissolved nutrients, and (3) all of this in response to daily to interannual differences in KPBS precipitation and temperature records (Nippert 2023).

A VELMA configuration (XML) file summarizes KPBS VELMA parameter values. Parameter definitions, associated state variables, process equations, and calibration methods are documented in the VELMA 2.0 user manual and supplemental user manual (McKane et al. 2014 and 2022). Table 1 summarizes key KPBS and other data sources used to calibrate and simulate VELMA plant, soil, and hydrological processes for KPBS and the Flint Hills ecoregion.

In summary, our top-down model calibration approach ensures that multivariate effects of climate, fire, grazing, and topographic variations in moisture and nutrients on ANPP and fuel loads are well integrated for spatially and temporally extrapolating observed KPBS ecosystem dynamics across the Flint Hills tallgrass prairie regional ecosystem.

For additional model calibration information, see Fig. 7 in the Results and Discussion subsection, “VELMA KPBS Data Synthesis for Flint Hills Extrapolation.”

### VELMA set up for simulating Flint hills ANPP and fuel loads

Extrapolation of the KPBS VELMA calibration for simulating Flint Hills ANPP and fuel loads involved the following 9 steps.

**Fig. 3:**
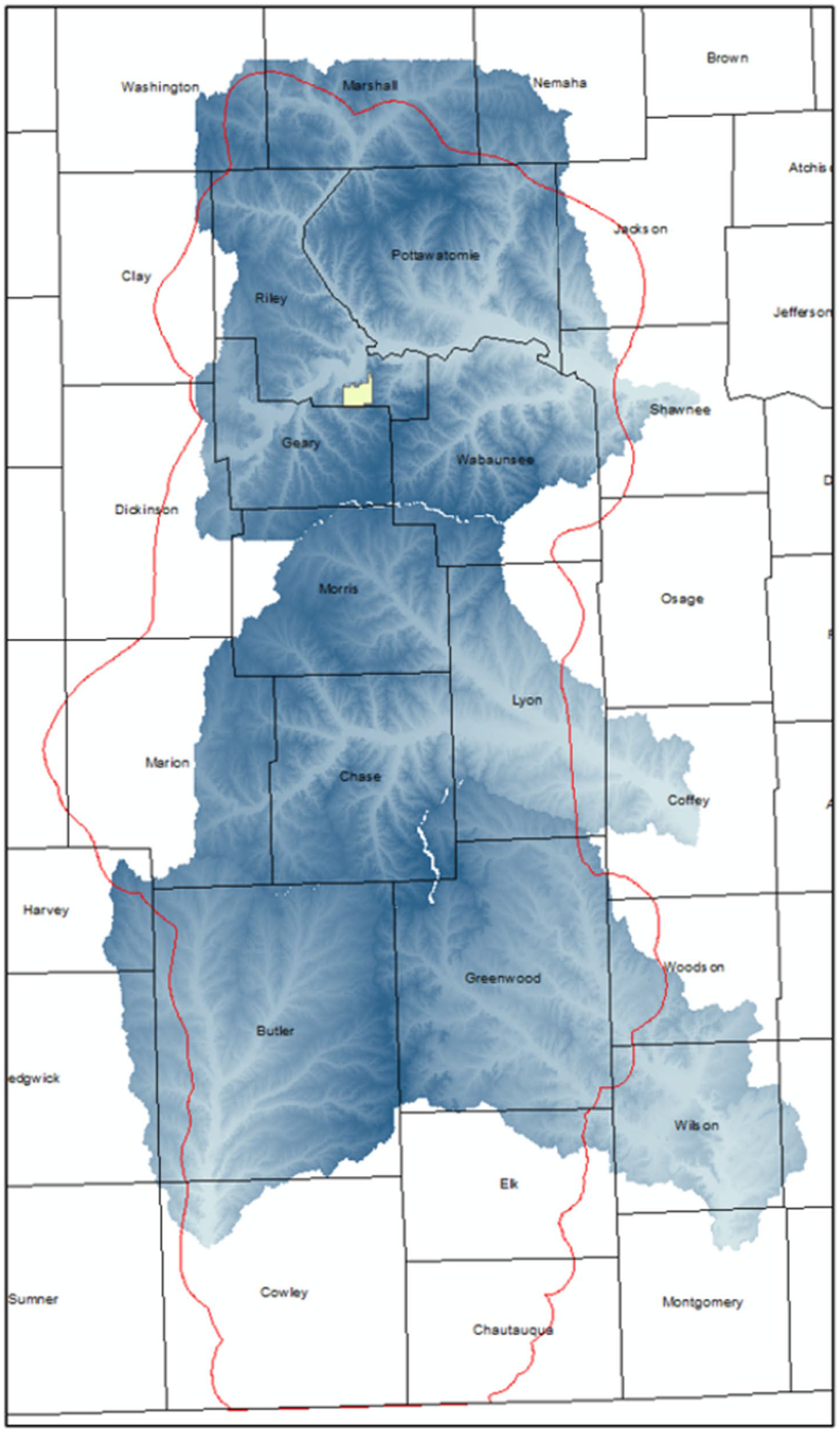
Flint Hills ecohydrological modeling domain. VELMA ▶ was applied to four regional watersheds—north, central, southeast, southwest—that together encompass most of the Flint Hills ecoregion (red line). The KPBS boundary is marked by the yellow polygon

**Fig. 4:**
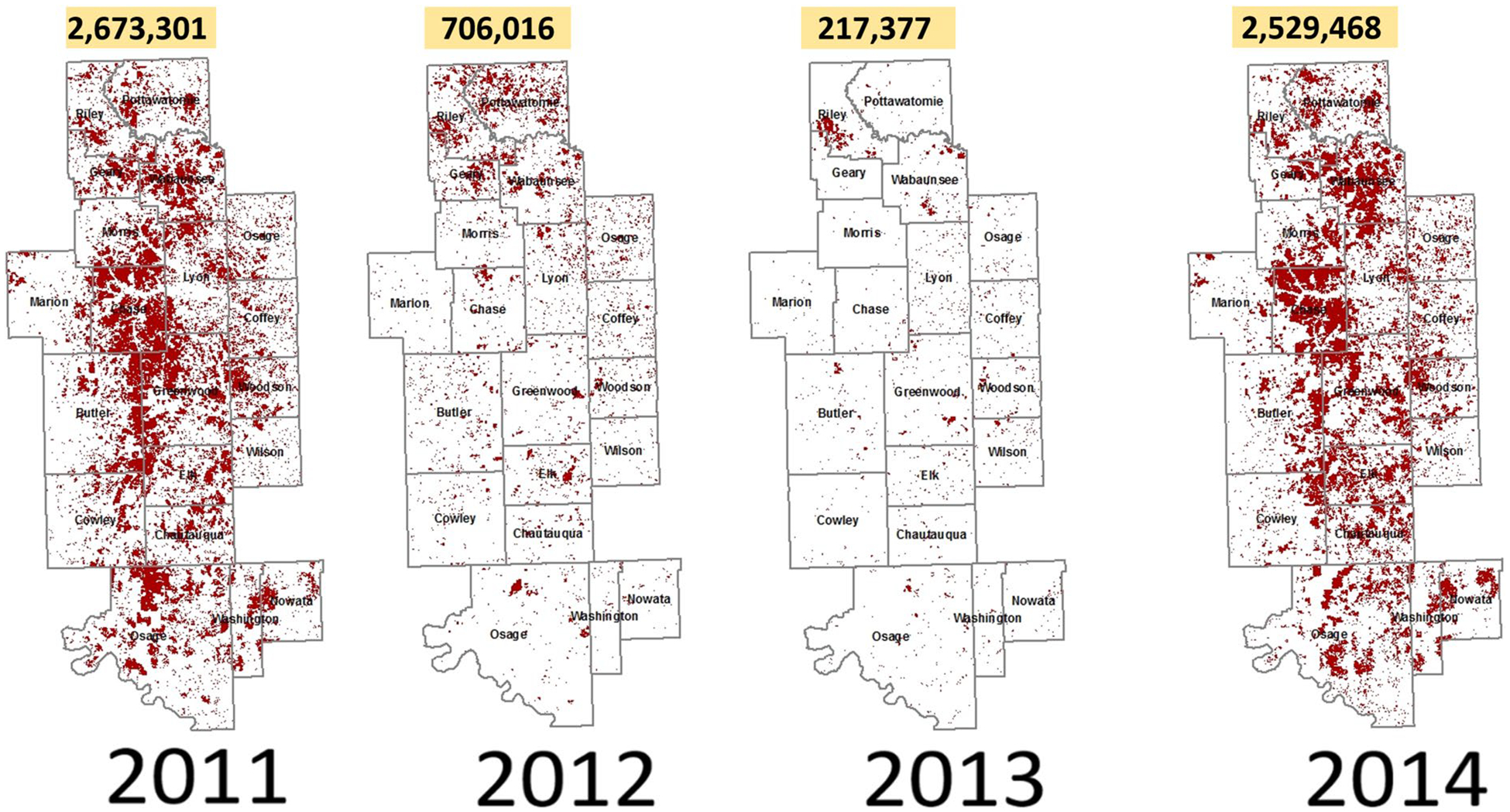
Flint Hills burn scars (red, acres burned/y) mapped for 2011–2014. 2012 and 2013 were drought years, showing significantly less prescribed burning. Source: Doug Watson and Jayson Prentice, Kansas Department of Health and Environment; Doug Goodin, Kansas State University

**Fig. 5:**
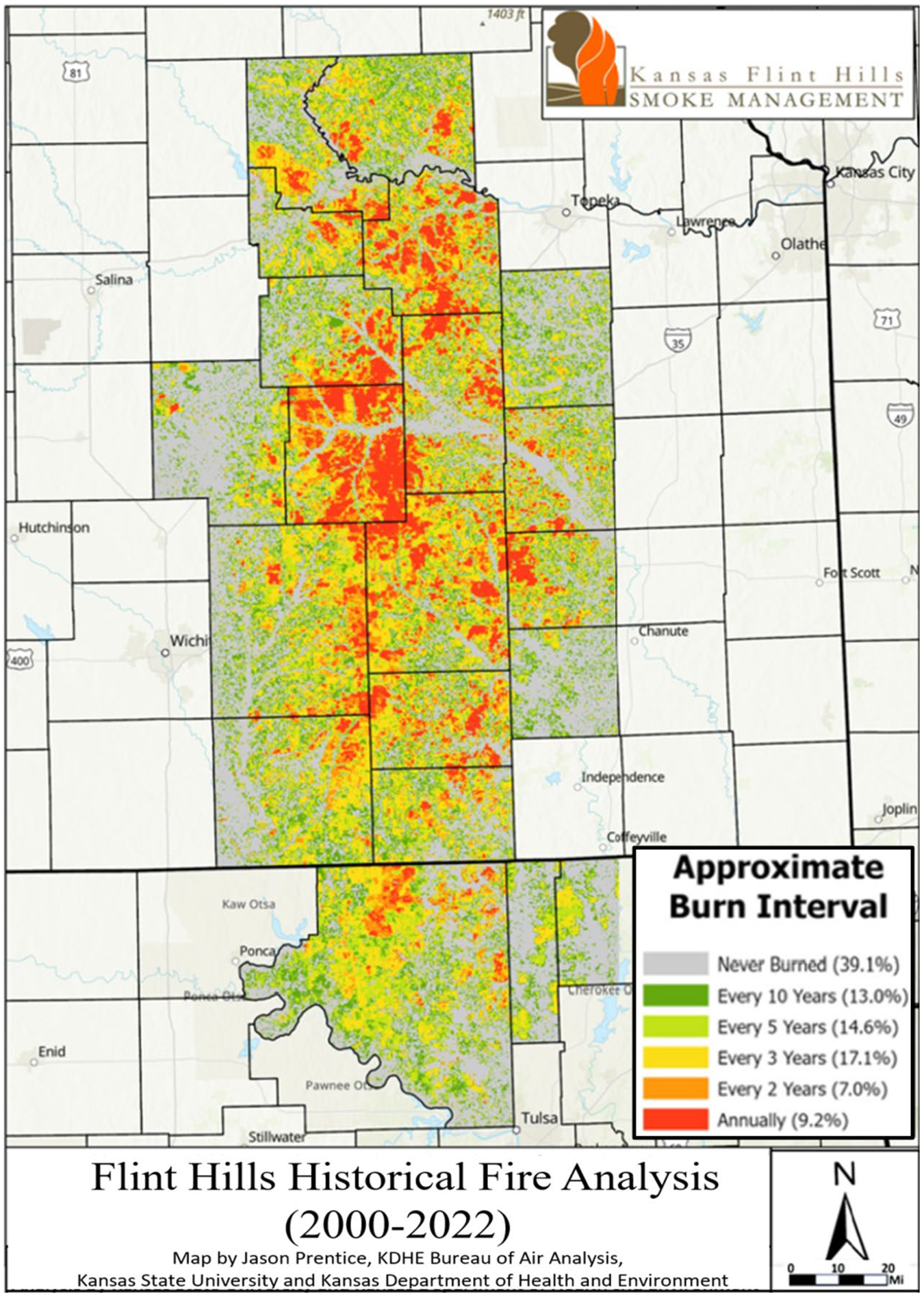
Flint Hills prescribed fire annual intervals detected through remote sensing technology. Of all land area that burned at least once during 2000–2022, 55% had a prescribed fire return interval of 3 years or less. Source: Jayson Prentice, Kansas Department of Health and Environment

**Fig. 6:**
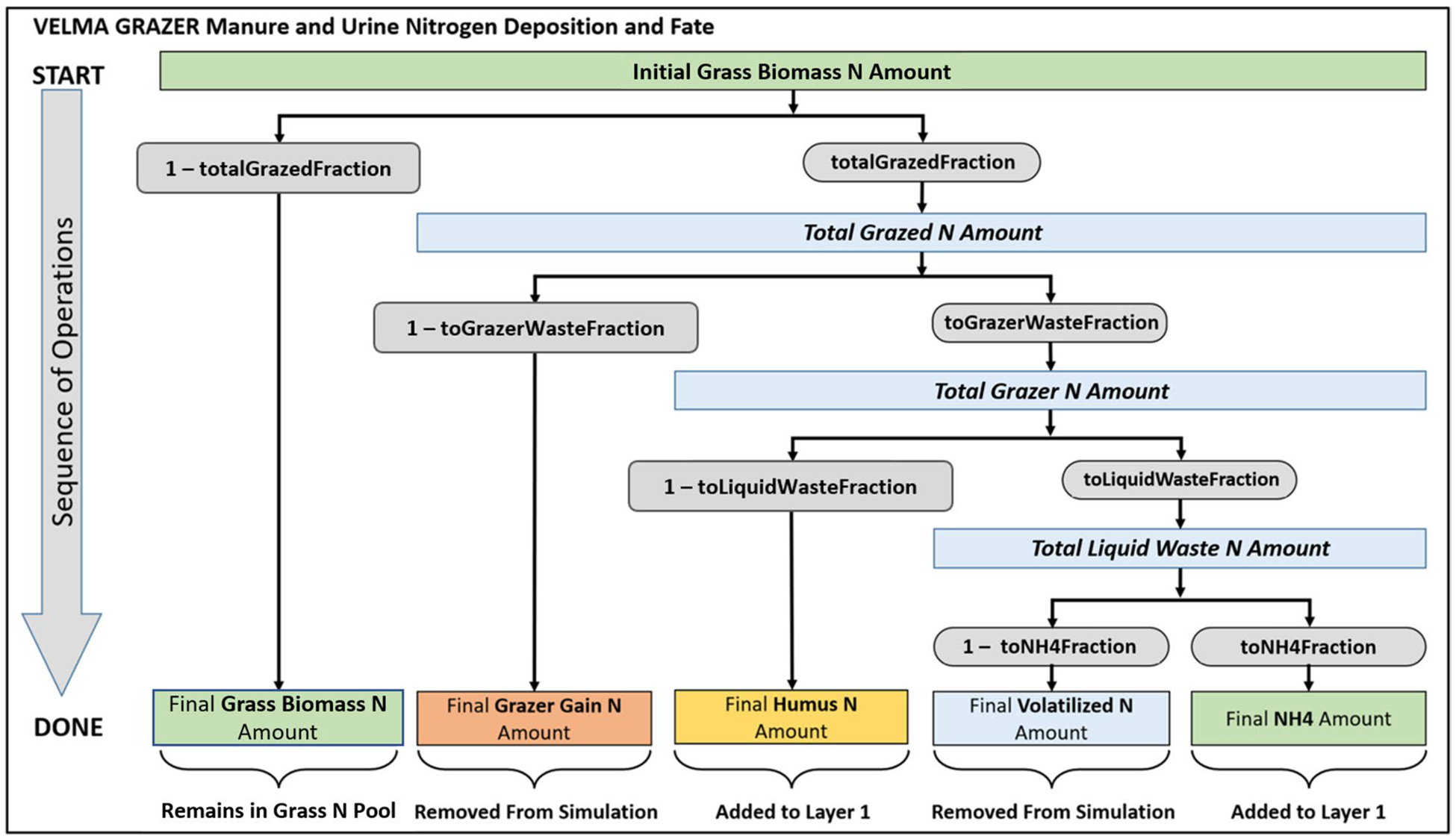
VELMA grazer nitrogen model describing the grazed fraction of standing live plant biomass N and its partitioning to grazer weight gain, manure (humus N), urine N, volatilized N, and soil ammonium N

**Fig. 7:**
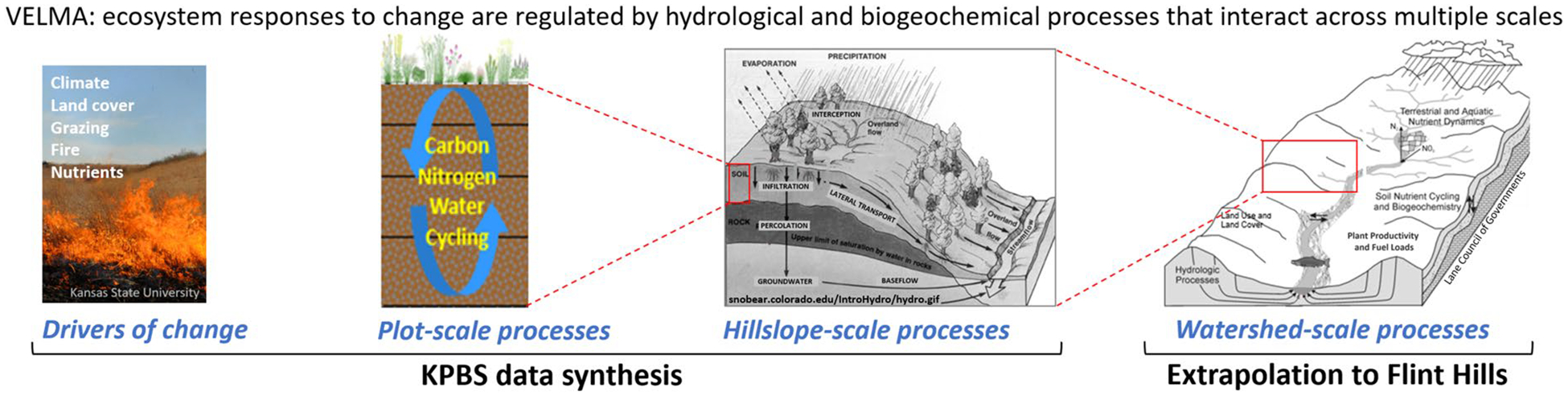
Visualization of the stepwise VELMA calibration approach used in this study for synthesizing multi-scale KPBS tallgrass prairie ecohydrological data for extrapolation to the Flint Hills ecoregion for informing rangeland management goals. See Table 1 in the Methods sections for key model drivers and references supporting VELMA’s KPBS data synthesis and its extrapolation to the Flint Hills

**Fig. 8:**
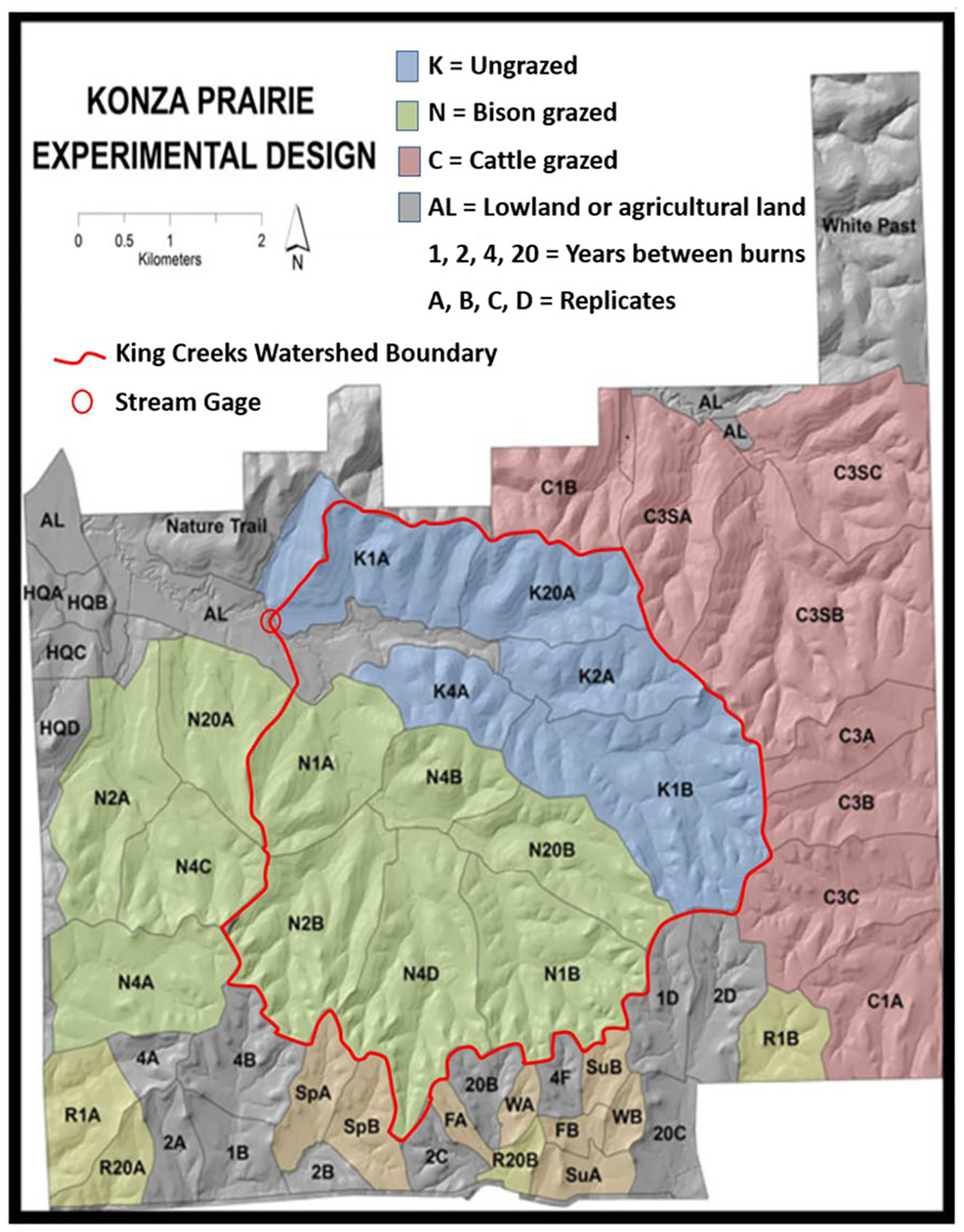
Konza Prairie Experimental Treatment Design. Source: Konza Prairie Long-Term Ecological Research Program

**Fig. 9:**
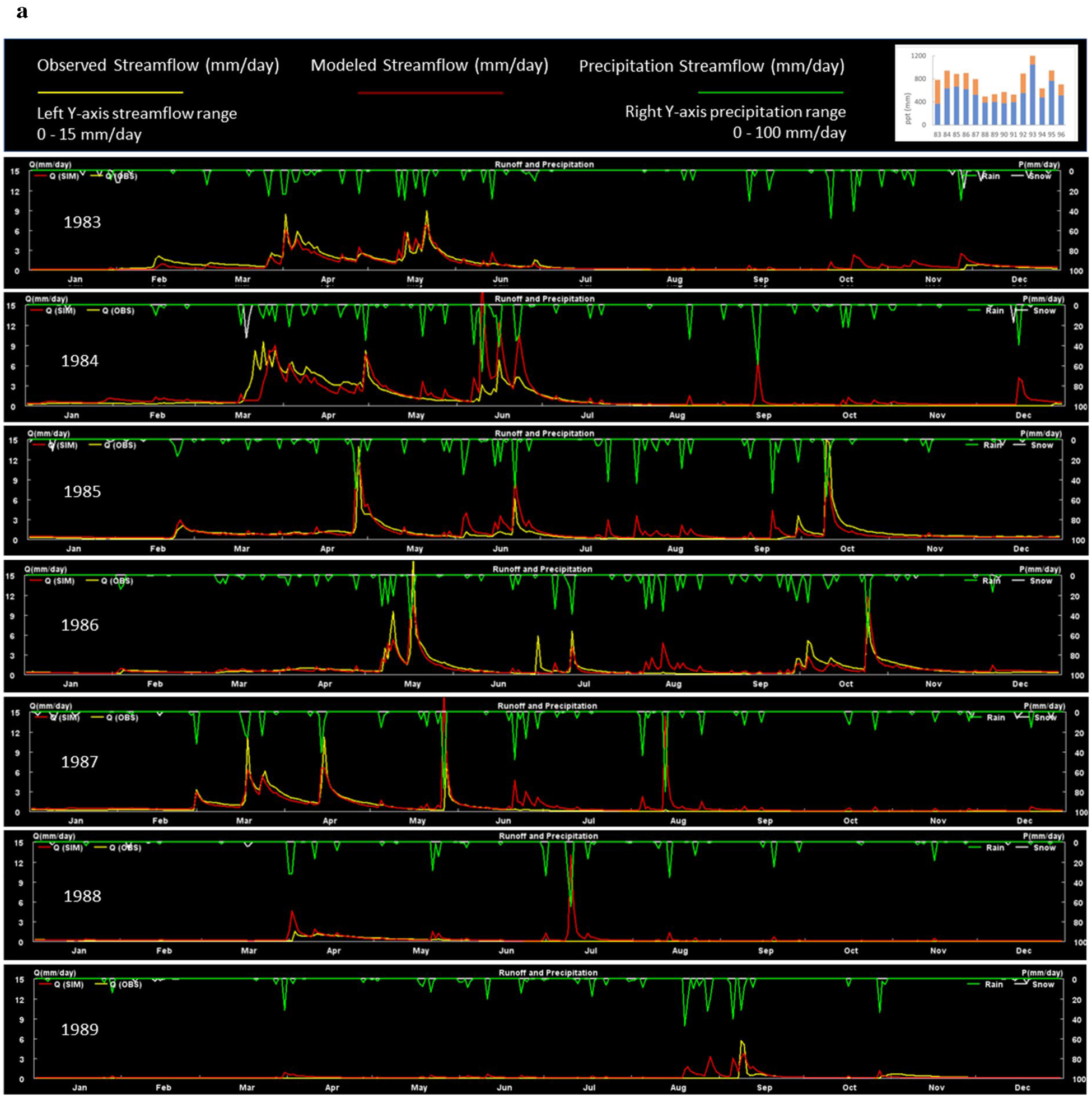

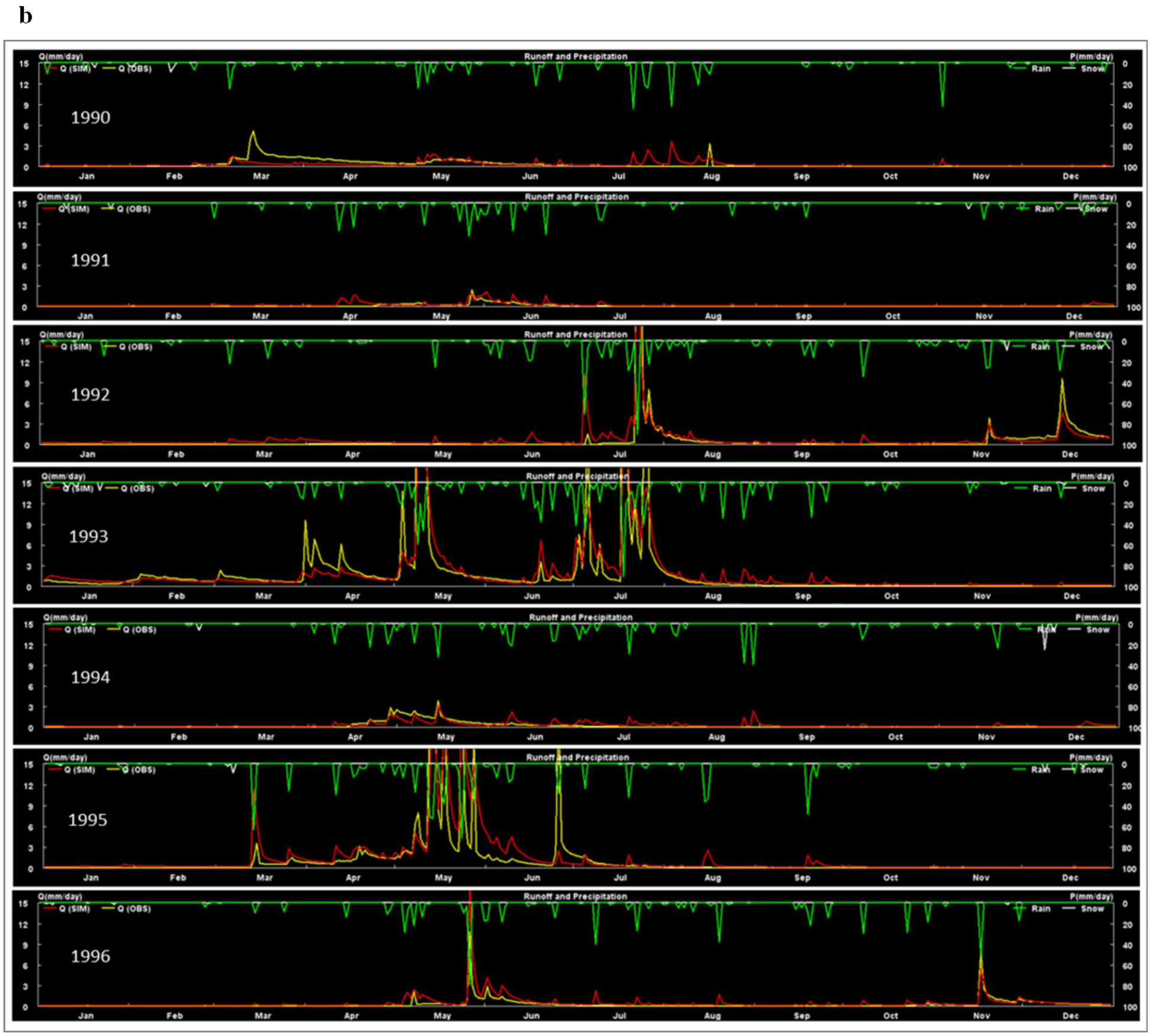
**a** VELMA simulated vs. observed daily streamflow at the Kings Creek watershed outlet for 1983–1989. Top right bar chart is the sum of annual and growing season precipitation (orange) for 1983 to 1996 (Nippert 2023). Streamflow for 1990–1996 is continued in Fig. b. **b** VELMA simulated vs. observed daily streamflow at the Kings Creek watershed outlet for 1990–1996. See Fig. a for legend details

**Fig. 10:**
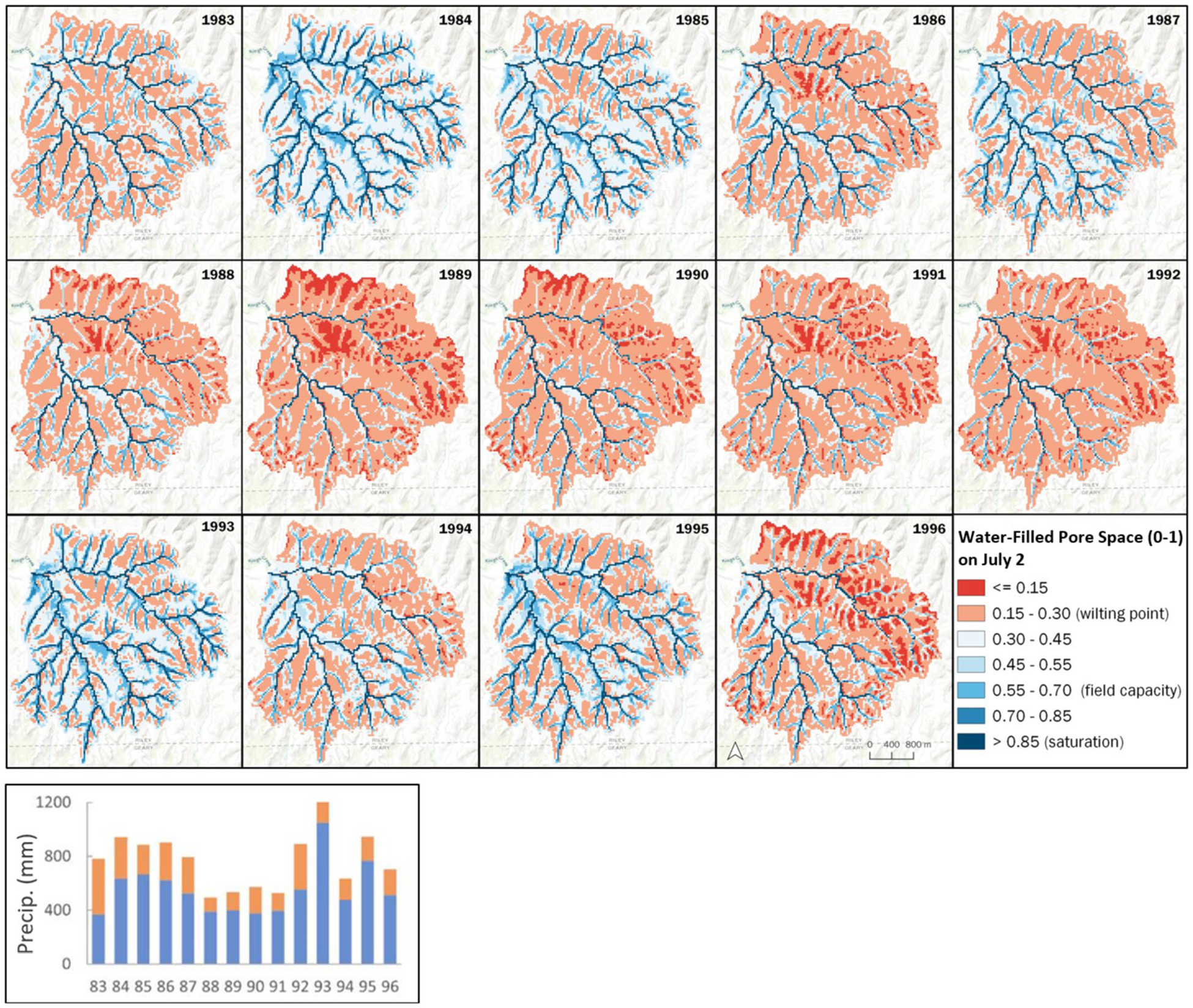
VELMA modeled soil water-filled pore space fraction (0–1) for KPBS Kings Creek watershed on July 2nd of every year from 1983 to 1996. Soil moisture conditions shown are whole soil column averages, not necessarily indicative of moisture conditions in each of VELMA’s 4 soil layers. Wilting point, field capacity, and saturation levels are based on Dunne and Leopold (1978) for KPBS soil textures (Ransom et al 1998). Lower right bar chart is the sum of annual and growing season precipitation (orange) for 1983 to 1996 (Nippert 2023)

**Fig. 11:**
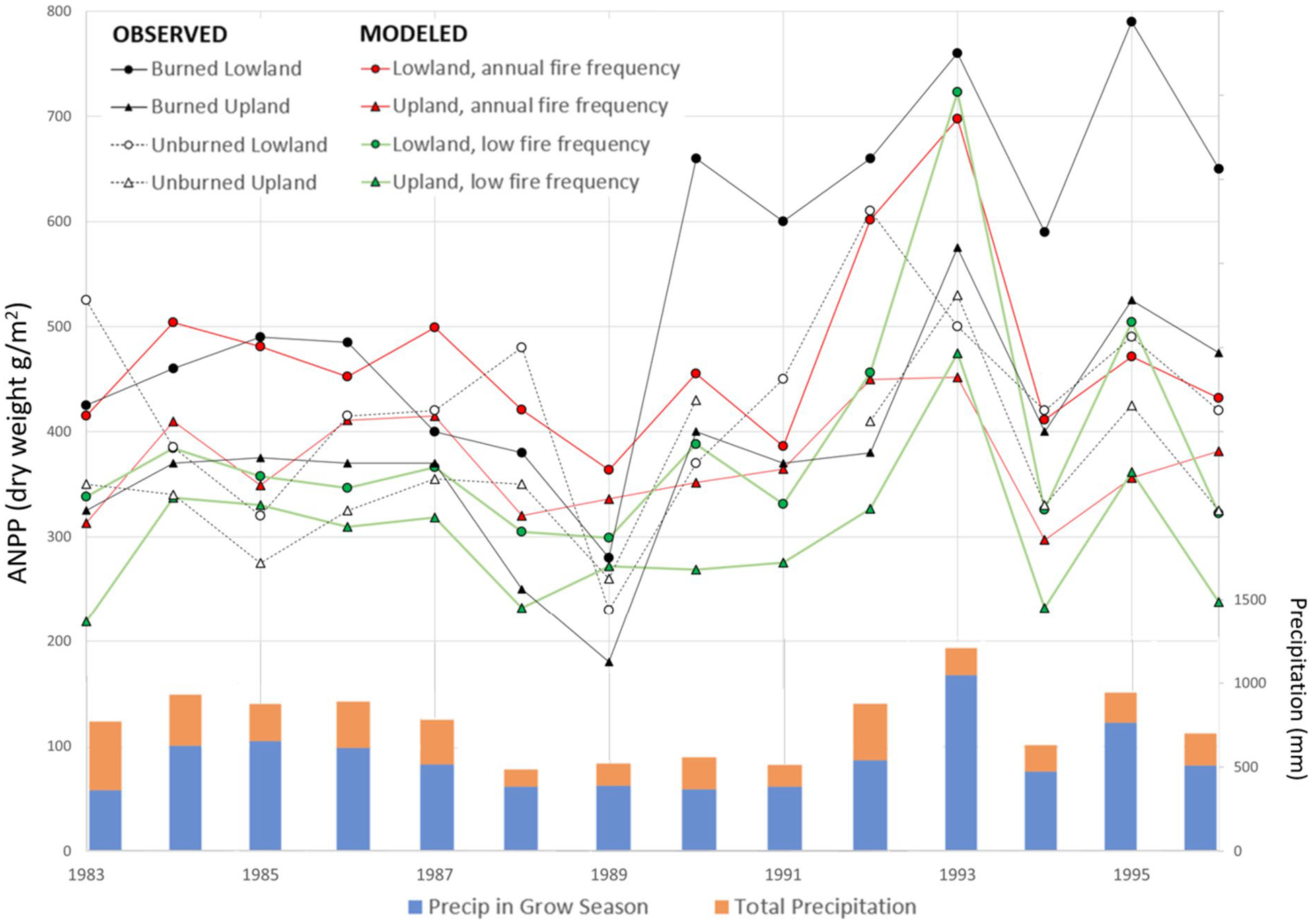
VELMA modeled vs. observed annual net primary production (ANPP dry weight g/m^2^/y) comparing ungrazed upland and lowland burned and unburned KPBS tallgrass prairie treatments. Observed ANPP data are from Briggs and Knapp (1995) and Knapp et al. (1998). Modeled ANPP data are for the KPBS Kings Creek watershed treatments marked in Fig. 8 for K20A = infrequently burned upland and lowland; and K1A and K1B = annually burned upland and lowland

**Fig. 12:**
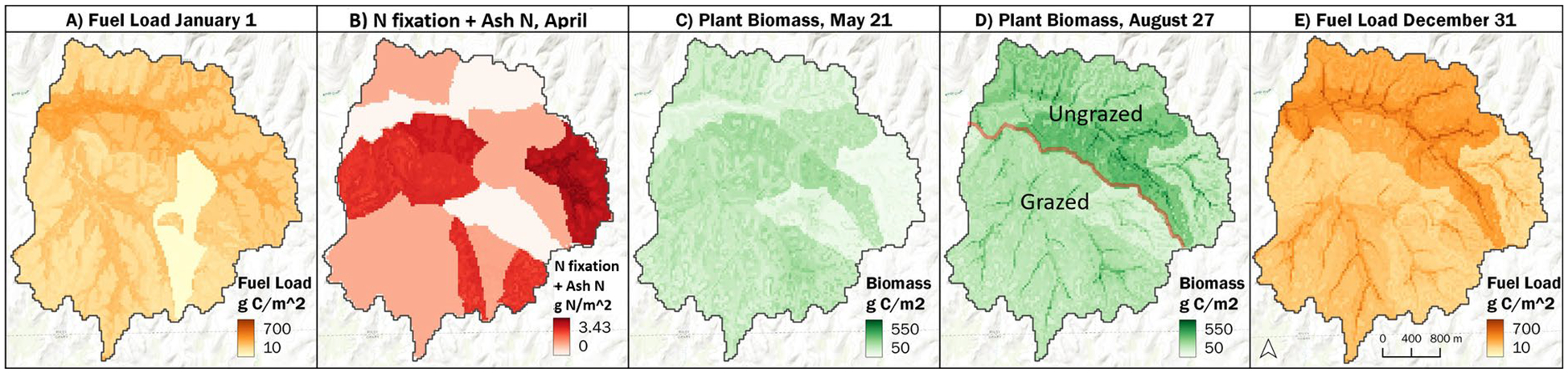
VELMA simulated Konza Prairie Kings Creek watershed 1993 time series: **A** initial fuel loads in January; **B** post-burn non-symbiotic N fixation + ash N in April; **C** aboveground plant biomass in mid-May; **D** peak-season aboveground NPP in August; and **E** end-of-year fuel loads. Frames B, C, D also highlight the combined benefits of non-symbiotic N fixation and ash N to spring and peak-season ANPP. In D, bison confined to the southern portion of the watershed grazed 20–25% of annual ANPP compared to the ungrazed northern (after their 1991 re-introduction to KPBS per Ratajczak et al, (2022)

**Fig. 13:**
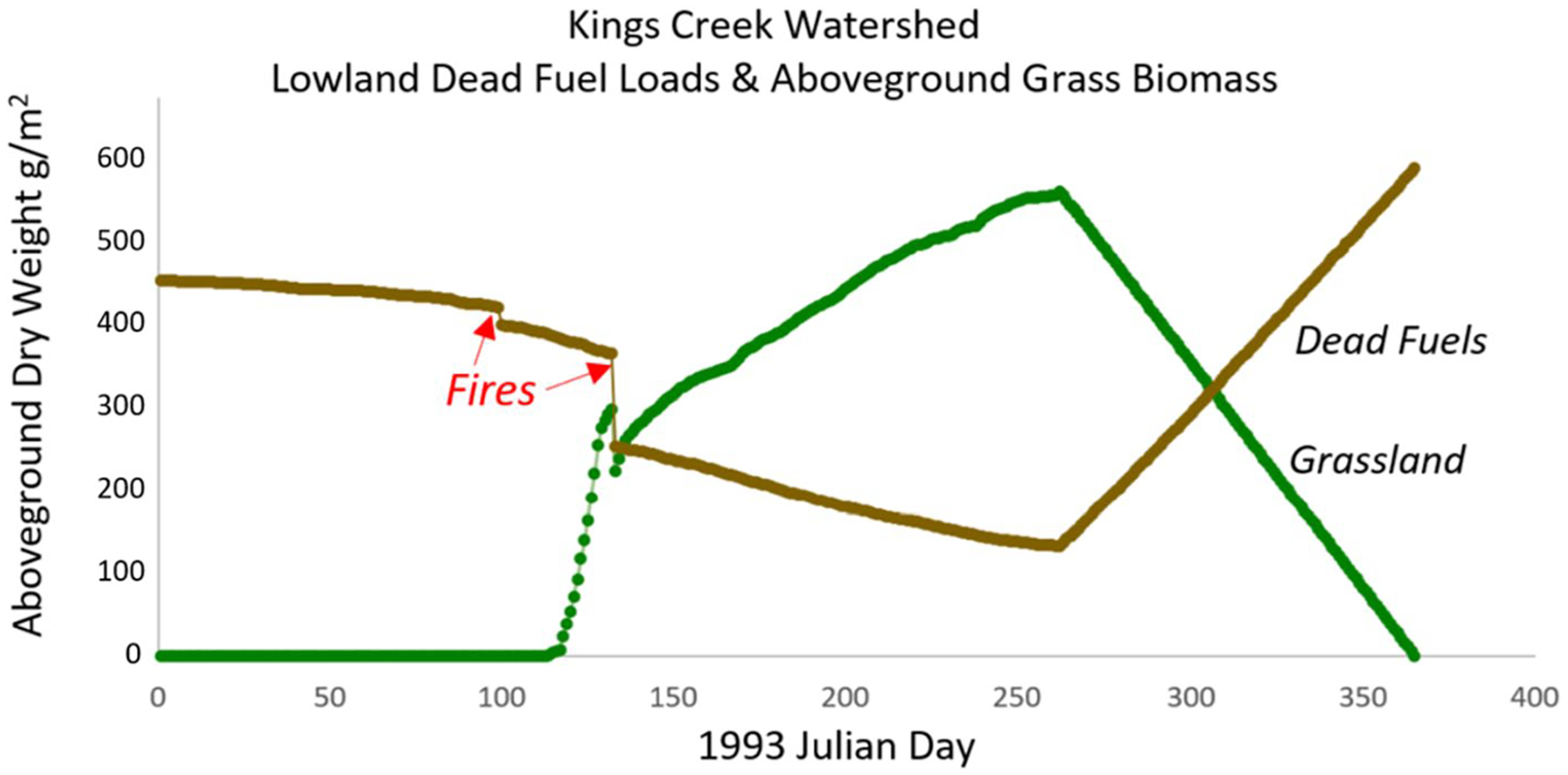
VELMA modeled 1993 dead fuel loads and aboveground grassland biomass (dry weight g/m^2^), averaged across lowland habitats for the 11.4 km^2^ KPBS Kings Creek water-shed. Treatments included grazed, ungrazed, burned and unburned tallgrass prairie treatments. Prescribed burns were conducted in various locations on April 9th and May 12th (Julian days 99 and 132). As noted in the caption for Fig. 1, the NLCD classification for grassland and pasture are both modeled herein as tallgrass prairie fire-managed ecosystems

**Fig. 14:**
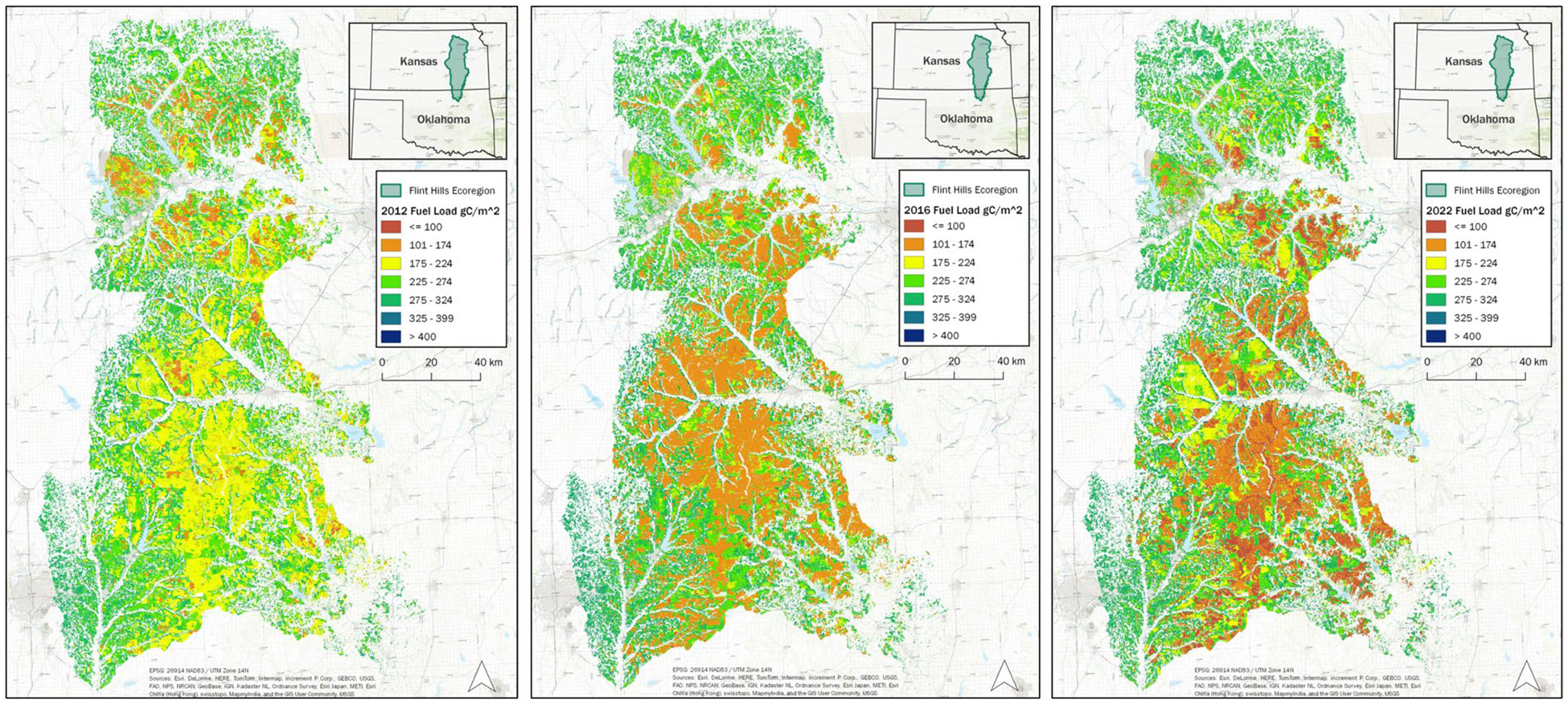
VELMA modeled Flint Hills ecoregion fuel load maps (g C/m^2^) for December 31 of 2012, 2016, 2022. These 3 years were chosen from maps developed for 2000 to 2022 to illustrate fuel load interannual variability. Non-grassland cover types (white grid cells on map) were modeled for hydrologic purposes but screened out as irrelevant to grassland fuel load mapping purposes. The Methods section describes modeling procedures to implement fire, grazing, weather data, and other details required to produce the Flint Hills fuel load maps

**Fig. 15:**
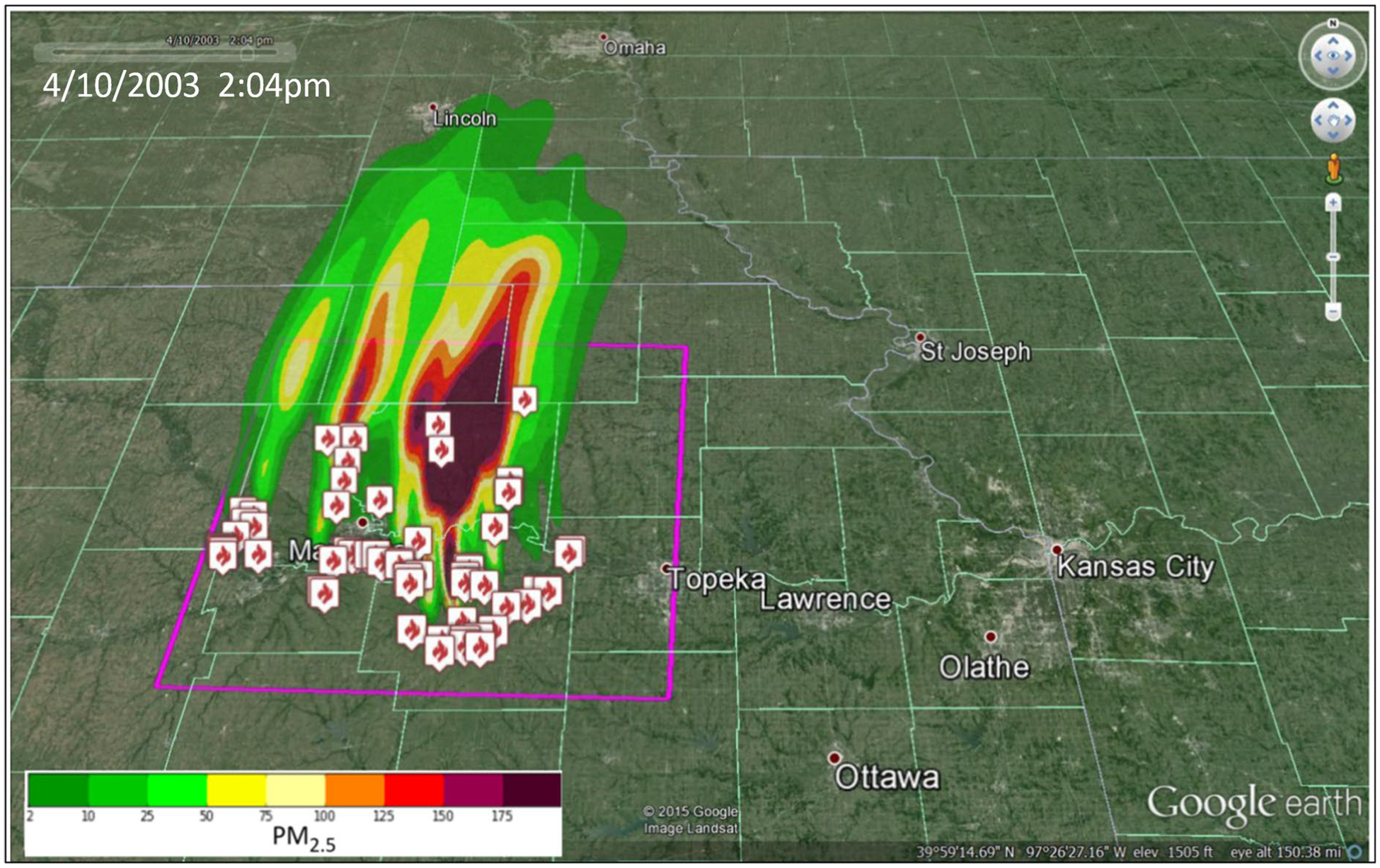
This BlueSky air quality model (Larkin et al 2009) demonstration shows simulated prescribed fire plume formation in the Flint Hills west of Kansas City on April 10, 2003, at 2:04 pm. Drivers for this simulation included VELMA fuel load estimates, MODIS day-of-burn prescribed fire locations, and weather drivers for extant conditions on April 10, 2003, at 2:04 pm (methods per Figs. 4 and 13 and Table 1). Modeled air quality constituents included particulate matter (PM_2.5_, shown), volatile organic compounds (VOCs), nitrous oxides (NOx), and ozone (O_3_). The purple box indicates the BlueSky Flint Hills simulation domain for highlighting near-source (white/red fire location symbols) and downwind plume PM_2.5_ concentrations

1. *Establish Flint Hills watershed boundaries, elevations, and flow paths*. We set up VELMA to include simulations of four watersheds draining in non-convergent directions that together encompass the majority of the Flint Hills ecoregion (Fig. 3). Watershed boundaries and cell-to-cell hydrologic flow paths were determined through application of the Java Digital Elevation Map (DEM) flat-processing tool, JPDEM (McKane et al. 2022, updated from Pan et al. 2012). Each VELMA 125-m grid cell has 8 neighbors (Fig. 2) with downslope cell-to-cell multi-direction flow proportionately weighted by elevation gradients.
2. *Establish grassland cover type boundaries*. National Land Cover Data (NLCD) were used to identify all tallgrass prairie grid cells within the Flint Hills for VELMA fuel load simulation purposes (Homer et al. 2004) (Fig. 1).
3. *Apply KPBS topographic soil properties to Flint Hills topography*. KPBS soil properties for VELMA-relevant physical and chemical soil properties were largely obtained from Ransom et al. (1998) and other coauthor contributors to Knapp et al. (1998). This included soil physical and chemical descriptions for soils on uplands, side slopes, foot slopes, terraces, and floodplains at KPBS. Key soil data included horizon and total soil depths, soil texture, and percent carbon. Corresponding soil N data were also obtained from other contributors to Knapp et al. (1998). This information was used to construct generalized Flint Hills soil profiles for these same topographic positions (Table 1).
4. *Map Flint Hills daily fire scars*. We used methods developed by Mohler and Goodin (2012) at the Kansas State University Remote Sensing Research Laboratory that involved analysis of Moderate Resolution Imaging Spectroradiometer (MODIS) satellite imagery to map the location and day of burned areas (fire scars) at a grid-scale of 250-m in the Flint Hills for each year from 2000 to 2022. Fire scar maps for each year were developed by Kansas State University and the Kansas Department of Health and Environment for VELMA Flint Hills applications. Figure 4 illustrates fire scar maps for four contrasting years. Figure 5 illustrates cumulative fire scars for all Flint Hills 250-m grid cells that burned at least once during 2000–2022. Approximately 55% of all cells managed with prescribed fire had a fire return interval of 3 years or less (Figs. 6, 7, 8, 9 and 10).
5. *Establish model scenarios for grassland burning*. KPBS burning scenarios used in VELMA were based on that site’s long-term prescribed burn treatments that specifies per parcel-specific burn intervals of 1, 2, 4, and 20 years (Fig. 8). Flint Hills grassland burning scenarios were simulated based on annual MODIS-derived date-of-burn fire scar maps (Fig. 4). This approach is consistent with standard rangeland management practices of conducting prescribed burning between during April–May, in advance of bringing in stocker cattle whose growth performance is enhanced by grazing the post-fire flush of grassland growth (Duncan et al. 2021). VELMA outputs for these KPBS and Flint Hills burning scenarios are essential for estimating effects on ANPP, fuel loads and, therefore, air quality impacts of burning (e.g., Results section Figs. 11, 12, 13, 14 and 15).
  Because MODIS fire-scar grids are scaled to 250-m grids, it was necessary to nest four VELMA 125-m grids within each 250-m grid to reduce documented effects of topography on grassland productivity (e.g., Briggs and Knapp 1995). Furthermore, considering the difference in grid scales between the KPBS VELMA calibration site (30-m) and our extrapolation of those model parameters to the Flint Hill ecoregion (125-m), we conducted a simple test to assess potential differences in modeled grassland ANPP for overlapping 30-m and 125-m grids across the same KPBS Kings Creek watershed location used to calibrate VELMA. Long-term studies at that watershed have established that ANPP is a sensitive indicator of the combined effects of climate, topography, and nutrient availability on grassland productivity. VELMA’s grassland annual NPP results for the two grids were very similar, with those for the 125-m grid always less than 3% higher during 1983 through 1994.
6. *Establish model scenarios for grassland grazing*. For Flint Hills applications VELMA grassland grazing scenarios were set up to interact with the grassland burning scenarios described in the preceding paragraphs. This grazing scenario simplification is based on the most common rangeland beef-cattle management practice in the Flint Hills. For that, grazing occurs on recently burned areas, beginning about 14 days after prescribed burning to take advantage of accelerated forage growth driven by improved soil nutrient availability and faster warming of sun-exposed soils. With this system, cattle grazing ceases 90 days later to allow late-season grassland recovery (removed cattle are typically transported to feedlots). Though there are some exceptions, the linkage of this grazing system to spring prescribed burning is applicable through most of the Flint Hills. We therefore set up VELMA Flint Hills grazing scenarios to be consistent with the timing and location of the burn scenarios described above.
  For our Flint Hills grazing scenario setup, VELMA’s grazer model grazing intensity parameter (daily fraction of aboveground grass biomass nitrogen grazed per grid cell) was set = 0.007 g N/m^2^/d for both bison and cattle. That value is based on long-term grazing data on KPBS experimental watersheds (Ratajczak et al. 2022).
  A map of KPBS grazing treatments is described in the Results and Discussion section (Fig. 8). Successful grazer model scenario performance tests are reported in the Results section for the southern portion of KPBS Kings Creek watershed, where resident bison graze year around (see Fig. 12 and subsection “Modeled Effects of Grazing on Grassland Biomass and Fuel Loads”).
  In addition to modeling grazing intensity, VELMA’s grazer model N budget accounts for deposition and cycling of N contained in cattle liquid and solid waste, and net N exported as weight gain with removed cattle. Fig. 6 summarizes VELMA’s grazer N budget (McKane and Barnhart 2017).
7. *Establish daily spatial climate drivers*. Flint Hills spatially distributed daily weather drivers for average temperature (average of minimum + maximum °C) and total daily precipitation (mm/d) were obtained for Daymet 1 km^2^ climate grids for years 1998 through 2018 (Daymet Version 4). Following a change in Daymet access protocols, PRISM daily climate data grids were used for years 2019 through 2022 (PRISM Climate Group 2022).
8. *Develop Flint Hills Fuel Load Maps*. Fuel maps were developed based on VELMA Fuel Load spatial outputs and 2001 NLCD data. For the four Flint Hills watersheds simulated (Fig. 3), VELMA calculated fuel load for every 125-m grid cell. For any given cell the resulting modeled fuel load values accounted for the daily effects of weather, fire, grazing, soil properties, and topographic effects on soil moisture. In GIS, fuel load biomass results from the four separately simulated watersheds were merged into a single spatial data layer, then adjusted to retain only cells corresponding to NLCD Class 71 Grassland/Herbaceous (Fig. 1) to create available fuel load maps for 2000–2022.
9. *Application of VELMA Flint Hills Fuel Load Estimates for Air Quality Modeling*. Numerous frameworks and modeling applications can utilize spatially explicit fuel load estimates to better understand impacts of land management on air quality and human health. For example, air dispersion and trajectory models—e.g., BlueSky (Larkin et al. 2009), AERMOD (Cimorelli et al. 2005)—can determine downwind air quality impacts, and full atmospheric chemistry and transport models—e.g., CMAQ (Byun and Schere 2006)—can simulate the production of ozone as well as its downwind trajectories. Combining fuel load estimates with these atmospheric models can help rangeland managers more accurately predict downwind air quality impacts of prescribed burning decisions and result in better short-term decision making (i.e., to burn or not to burn) on any given day during a burn season. While a version of this has already been implemented at the county-scale (see www.ksfire.org) using qualitative point-based estimates by rangeland managers, the process-based fuel load simulations developed here provide more temporally resolved and spatially explicit estimates over the entire Flint Hills region. When looking at longer timescales of days to decades, these improved modeling frameworks may also be combined with human health and economic impact models—e.g., BenMAP-CE (Sacks et al. 2018)—to assist in the coordinated decision-making among all Flint Hills stakeholders to understand, estimate, and balance ecological and air quality trade-offs for actual and hypothetical differences in the location, timing and frequency of rangeland fires.

## Results and discussion

Our main objectives were to (1) use VELMA to conduct a performance-tested synthesis of long-term KPBS experimental data capable of describing the interactive effects of climate, soil moisture, topography, N and C dynamics, fire, and grazing, on tallgrass prairie productivity and fuel loads; and (2) spatially and temporally extrapolate VELMA’s KPBS data synthesis for estimating productivity and fuel loads across the Flint Hills tallgrass prairie ecoregion in eastern Kansas, USA, with the goal of supporting smoke dispersion and trajectory modeling. Consistent with this multi-scale analysis, we present the KPBS data synthesis first and its regional extrapolation for Flint Hills air quality modeling second.

### VELMA KPBS data synthesis for Flint hills extrapolation

Results for the following VELMA KPBS data synthesis subsections followed a stepwise VELMA calibration approach to simulate how climate and management drivers influence watershed hydrologic processes, and how those effects in turn influence biogeochemical processes affecting grassland productivity and fuel loads across small and large spatial scales. The calibration methods used are described in broad outline in the Methods section.

These multi-scale ecohydrological interactions and their consequences are complex and challenging to communicate. Here we combine the results and discussion of this approach into a more concise, readable narrative rooted in systems thinking, making it easier to highlight interconnected processes, feedbacks, and emergence of predictable landscape patterns.

For discussion purposes, Fig. 7 is a more intuitive visual summary of our VELMA KPBS data synthesis calibration approach that augments calibration information presented earlier in the Methods section. This figure’s visual conceptualization emphasizes how we used VELMA to conduct a stepwise synthesis of multi-scale KPBS ecohydrological data, and how we extrapolated that synthesis to the nearly 1,000 times larger Flint Hills tallgrass prairie ecoregion.

More specifically, we *sequentially calibrated* VELMA to model how various drivers of change affect six KPBS ecohydrological responses: streamflow, soil moisture, NPP, grazing, fuel loads, and effects of fire on air quality. Importantly, this stepwise calibration approach provided a means for assessing (1) how each of these six model results in sequence is tightly linked to those before it; and (2) how, for discussion purposes, the KPBS integration of these modeled ecosystem responses enabled their extrapolation across scales ranging from small plots to hillslopes to the hill-and-valley topography characteristic of local watersheds across the Flint Hills ecoregion.

We present Fig. 7 here rather than at the end of the Methods section to facilitate our discussion of the VELMA KPBS and Flint Hills results that follow, including how the processes modeled at each step (subsection) represent a process-based extension from the preceding subsections, culminating with a VELMA-BlueSky visualization in the last section describing how prescribed rangeland fires in a central Flint Hills location can impact downwind air quality.

### Modeled KPBS streamflow results

KPBS research points to soil moisture as an important variable that can directly limit grassland productivity, and does so indirectly through constraints associated with ecosystem N cycling. Therefore, because hillslope and watershed-scale patterns of soil moisture are runoff dependent, calibration of VELMA to simulate watershed-scale streamflow was step one to its KPBS implementation.

We used high quality daily streamflow data (Dodds 2023) to calibrate VELMA for the 11.4 km^2^ Kings Creek watershed, located entirely within the KPBS boundary (Fig. 8). Kings Creek is a fourth-order creek that drains a mixed agricultural, pasture, and tallgrass prairie dominated watershed. Quoting Mast and Turk (1999): “Kings Creek is an intermittent stream with sustained flows generally occurring in the spring. Average annual precipitation is about 84 cm, which mostly falls in the form of rain during spring and early summer. Streamflow generally ceases during late summer, and the stream may remain dry during the fall. Flow in ephemeral headwater channels occurs only immediately following rainfall events and average annual runoff is about 20 cm (Putnam et al. 1996).”

As shown in Fig. 8, KPBS managers established long-term experimental burning and grazing treatments that follow subwatershed boundaries within the Kings Creek watershed, enabling researchers to study process-level effects of grazing, prescribed fire, and topography on streamflow, soil moisture, plant productivity, fuel loads, and many other ecosystem properties.

Using 30-m grid cells to model the Kings Creek watershed (Table 1), VELMA generally captured observed streamflow responses to storm events and droughts. However, depending upon antecedent drought conditions—for example, during the summers of 1988–1991—VELMA tended to overpredict corresponding Kings Creek streamflow responses to rainfall events (Fig. 9a, b). This modeled behavior is consistent with a hydrologic analysis of the Kings Creek watershed by Costigan et al. (2015). They reported many instances of precipitation and flow desynchronization between headwater streams and the Kings Creek mainstem. Their analyses indicate that headwater catchment hydrologic processes, such as groundwater storage in limestone and alluvial strata, and dynamic infiltration flow paths within those strata, produce a threshold-driven hydrologic response that decouples headwater and mainstem flow responses to precipitation. VELMA cannot capture these dynamics because it does not model deep groundwater (aquifer) flow dynamics, instead being restricted to soil survey depths.

That said, there are hints within VELMA’s streamflow data that deep groundwater dynamics may be at play during low rainfall years when groundwater threshold responses would most likely occur (Costigan et al. 2015). Those hints are in VELMA streamflow predictions during the very dry growing seasons of 1989 and 1990 (Fig. 9a, b). Specifically, a series of modest mid-year rainfall events occurred in August–September 1989, and July–August 1990. For both times, VELMA simulated intermittent streamflow spikes while observed streamflow was flatlining. After several such precipitation events spread over about one month during each year, observed streamflow displayed one spike at the end of VELMA’s series of false spikes. We hypothesize that lateral downslope flow in VELMA’s near-surface soil layers is much faster than actual infiltration flow paths through deep limestone/shale/alluvial strata prone to threshold-driven discharges (Costigan et al. 2015).

Our main purpose in calibrating VELMA to simulate Kings Creek streamflow dynamics was to accurately constrain soil moisture levels that, in turn, potentially constrain tallgrass prairie net primary production. Even without a deep groundwater component for VELMA, its current Kings Creek streamflow predictions suggest a sufficiently good fit to observed data to support these objectives.

### Modeled KPBS soil moisture results

Soil moisture levels in watersheds reflect the combined effects of precipitation totals and timing, evapotranspiration, and topographic position on runoff and soil moisture. Figure 10 describes VELMA modeled patterns of soil moisture for Kings Creek watershed on July 2nd of each year during 1983 to 1996. We chose July 2nd to compare interannual patterns of soil moisture during 1983–1996 because plant water stress evident at that growing season mid-point can be a strong determinant of peak aboveground net primary production, typically occurring around late August. This span of 14 years coincides with observed peak ANPP data for various KPBS tallgrass prairie management treatments (Briggs and Knapp 1995; Knapp et al 1998).

VELMA soil moisture results in Fig. 10 are expressed as the fraction (0–1) of water-filled pore space (WFPS). Modeled values are averaged across all 4 soil layers per 30-m grid cell. Soil moisture values at or below plant wilting point (WFPS < = 0.3) are highlighted in red. This threshold corresponds to values reported for silty clay and silty clay loam soils (Dunne and Leopold 1978) typifying KPBS soils (Ransom et al. 1998). WFPS fractions above wilting point are shown in intensifying shades of blue up to 1.0 at saturation.

In Fig. 10, the most important spatial and interannual patterns of modeled July 2nd soil moisture are generally associated with (1) topography, such that upland soils are always significantly drier than lowlands; and (2) low vs high precipitation years. For example, 1988 was the driest year during 1983–1996, yet did not have the lowest July 2nd soil moistures, having been preceded by the relatively wet year 1987. 1989 and 1990 by comparison received slightly more precipitation than 1988 but became progressively drier as persisting drought conditions depleted soil moisture reserves into 1990. By contrast, 1993 received near record precipitation, with July 2nd soil moisture levels exceeding all other years except 1984.

Note that because July 2nd moisture patterns shown in Fig. 10 are an average for VELMA’s four soil layers, any one layer above wilting point could provide drought relief. Modeled soil moisture effects on ANPP are discussed after the next section.

### VELMA model constraints on tallgrass prairie ANPP

VELMA simulates how seasonal and interannual variations in the availabilities of soil water and N can interchangeably limit NPP in time and space within watersheds, thereby providing a way to explore the hypothesis that spatial and temporal patterns of vegetation productivity may best be explained within the context of a nonequilibrium system where the relative constraints of N, water, and temperature vary in both space and time (Seastedt and Knapp 1993; Blair 1997).

Consistent with this idea, VELMA simulations impose four potential constraints to plant uptake of NH_4_ and NO_3_ and, therefore, NPP:

(1) $$NPP\;g\;N/m^{2}/d=RBN*\text{TNUscalar}*NMU*(\text{Nscalar}\;or\;\text{Wscalar})$$

where, RBN = Root biomass g N/m^2^ (Knapp et al. 1998), distributed vertically by soil layer (Jackson et al. 1996).

TNUscalar = Temperature N uptake scaler (0–1) for the effect of soil temperature on plant N uptake (Rastetter et al 1991; McKane et al. 1997) calibrated for tallgrass prairie.

NMU = Nitrogen maximum uptake potential by plants (g N/m^2^/d) calculated for tallgrass prairie based on soil N and plant NPP data (Knapp et al. 1998) and Michaelis-Menton uptake kinetics for NH_4_ and NO_3_ (e.g., Kuzyakov and Xu 2013).

Nscalar *or* Wscalar = nitrogen scalar or water scalar, each ranging 0–1 and calculated by VELMA based on simulated daily plant available soil N and soil H_2_O, such that VELMA selects the scalar having the lowest value to reduce NMU per soil layer per 30-m grid cell.

For example, if Nscalar = 0.2, and Wscalar = 0.6, then Nscalar * NMU * TNUscalar * RBN = NPP g N/m2/d, wherein NPP can be converted from units of N to C and thence dry weight units, after making the appropriate C/N and dry weight/C conversions. Derivation of VELMA’s NPP equation can be found using Rastetter et al. (1991) and McKane et al. (1997).

### Modeled vs. observed interannual variability in tallgrass prairie ANPP

Figure 11 is a summary of KPBS modeled vs. observed ANPP data reported for 1983–1996 by Briggs and Knapp (1995) and Knapp et al. (1998). This comparison includes the four KPBS experimental treatments reported by those authors: burned and unburned upland, and burned and unburned lowland. All four treatments are ungrazed. The Fig. 11 caption describes where the modeled treatments are located within the KPBS Kings Creek watershed treatment map (Fig. 8).

To obtain modeled annual ANPP during 1983–1996 for each of the four treatments, VELMA’s geospatially mapped results for peak-season aboveground plant biomass were sampled on August 27 of each year across multiple sentinel locations per treatment using GIS methods (QGIS 3.28, QGIS Association). Values for VELMA peak ANPP g N/m^2^ per treatment year were converted to g dry weight/m^2^ per treatment year for comparison to the Briggs and Knapp observed data in Fig. 11.

#### Interannual ANPP differences across treatments

Figure 11 shows a close but imperfect correspondence between modeled and observed ANPP across the burned and infrequently burned upland and lowland treatments. Henceforth, “infrequently burned” = “unburned”, following Briggs and Knapp (1995). Across all treatments, observed and modeled ANPP spanned a range of about 200 to 800 g/m^2^. At the low end of the ANPP range were *upland unburned* treatments, with modeled values closely tracking observed ups and downs during 1983–1992, then falling below observed values during 1993–1996 while still tracking ups and downs in observed trends.

At the high end of the ANPP range, observed and modeled *lowland burned* treatments generally exceeded all other treatments, with highest values between 500 and 800 g/m^2^, and with modeled values generally tracking observed peaks and valleys to within 50 to 100 g/m^2^, with some exceptions at the highest observed values.

Observed and modeled lowland unburned ANPP values were generally next highest, not far below their lowland burned treatment counterparts. Significantly, ANPP of all treatments declined in 1989, many to less than half their highest levels across all years. 1989 was the second driest year, following the driest year during this 14-year study.

#### Soil moisture constraints on ANPP

In summary, across years and treatments, VELMA captured general trends in the observed effects of topographic position and fire on ANPP. Most importantly, the modeled results provide process-based insights into observed data for the four treatments describing ANPP responses to fire and topographic position. Considering topographic effects first, Fig. 10 watershed-scale patterns of soil moisture illustrate how topography and interannual differences in precipitation combined to create extreme spatial and temporal differences in water stress by mid-growing season (always July 2nd in Fig. 10). The color shading in Fig. 10 indicates areas of high and low water stress within the Kings Creek watershed during certain years: red = moisture at or below plant wilting point; light to dark blue = moisture increasing toward field capacity and saturation. The sharp declines in observed and modeled ANPP for most treatments during 1988–1989 correspond to declining soil moisture levels, especially in 1989 when modeled soil moisture reached or approached wilting point across uplands, hillslopes, and some lowland stream terraces.

An important Fig. 10 takeaway is that across all upland treatments, those having the highest ANPP (Fig. 11) include locations where July 2nd modeled soil moisture levels are near or below plant wilting point (red tones). The longest such dry period occurred during 1988–1992, with 1989 being the driest. Comparing the Fig. 10 red soil moisture zones to Fig. 8 treatment locations, and to corresponding Fig. 11 ANPP levels, affirms that high ANPP (and leaf biomass) in VELMA corresponds to high water use (evapotranspiration, ET) that intensifies drought conditions. This ET effect is most intense on upland soils, with some degree of drought relief in hillslope drainages. As shown later in Fig. 13, most modeled gains in ANPP (~ 70%) and associated ET losses occur before July 2nd, helping to explain the somewhat counterintuitive correlation between high ANPP and dry soils, at least for upland locations.

In contrast, lowland soils generally maintained high soil moisture levels in areas immediately surrounding Kings Creek, though less so on more elevated stream terraces. Higher ANPP values for lowland vs upland reflect the spatial patterns of water stress in Fig. 10. This topographic effect is especially pronounced for the years when water stress has deepened by July 2nd through a combination of low precipitation and high ET.

#### Precipitation timing and ANPP

As mentioned above, 1989 had extremely low soil moisture levels on July 2nd, having just followed the lowest growing season precipitation year in this 14-year precipitation record, 1988. This appears to explain the 1989 crash in ANPP across all treatments (Figs. 10, 11). Whereas 1990 soil moisture conditions appear almost as dry as 1989, ANPP rebounded sharply in 1990, despite only a slight increase in 1990 growing season precipitation compared to 1989. The explanation for this counterintuitive 1990 all treatment ANPP rebound is in the daily precipitation and stream hydrographs for 1989 and 1990. 1989 growing season precipitation arrived late, falling during mid-August to early September (Fig. 9a), too late in the growing season to appreciably influence ANPP. In contrast, 1990 received most of its growing season precipitation over a month earlier (Fig. 9b), during early July through early August—early enough to reduce water stress and support the strong all-treatment ANPP rebound seen in both modeled and observed ANPP (Fig. 11). Recall that Fig. 10 does not display soil moisture after July 2nd, so the dynamic of later vs earlier precipitation is not apparent there.

Also of note in Fig. 10 is that grazing by resident bison in the southern portion of the Kings Creek watershed (“N” treatments in Fig. 8) kept aboveground biomass levels about 20–25% below ungrazed “K” treatments to the north, thereby keeping ET water losses lower and soil moisture higher (Ratajczak et al. 2022).

#### Nitrogen constraints on ANPP

Fire is the other treatment factor leading to major observed differences in ANPP, with burned lowland ANPP at times exceeding that of unburned lowland by up to 70% after 1990 (Fig. 11). VELMA’s capabilities for modeling and mapping time series of ecosystem C and N interactions provided insights into post-fire increases in ANPP that are not readily apparent with observed data alone.

For the Kings Creek watershed, modeled post-fire increases in ANPP were primarily associated with the combined effects of increased soil nitrogen fixation (Eisele et al. 1989); smaller amounts of ash N deposition (Hobbs et al. 1991); and the slow but steady release of N from decomposing surface fuels and soil organic matter. Figure 12 maps how these grassland N cycling processes interacted during the course of 1993 to produce changes in ANPP and fuel loads.

Figure 13, described in the final Results and Discussion section in the context of watershed and ecoregion fuel load and plant biomass dynamics, provides a graphical daily timeline take on Fig. 12. It describes the daily dynamics and interplay of (1) fuel load N; (2) the post-fire combination in N fixation + ash N leading to short-term stimulation of ANPP; (3) the subsequent ANPP slowdown as soil N and/or water stress increase through the summer; and (4) the gradual transfer of live biomass N to surface fuel N during plant senescence. A key theme here and throughout the present section is that the modeled spatial and temporal interplay of N and water constraints on ANPP tends to vary seasonally and interannually.

#### Variable constraints on ANPP

To sum up, our model-based interpretations of Fig. 11 observed ANPP results benefited from process-based insights into underlying water and N constraints to ANPP (Fig. 9, 10, 12 and 13). In doing so, VELMA’s synthesis of diverse KPBS datasets has extended those data by allowing interactions among key ecosystem-scale variables—soil moisture, available soil N, NPP, and fuel loads—to be simulated and mapped for evaluating spatial and temporal changes in water and N constraints on NPP and ecosystem carbon dynamics. These model-based insights are consistent with the transient maxima hypothesis, postulating that patterns of productivity in tallgrass prairie are best explained within the context of a nonequilibrium system for which the relative constraints of N, water, and energy that limit ecosystem dynamics tend to vary in both space and time (Seastedt and Knapp 1993; Blair 1997).

Taken together, the VELMA results and concepts presented to this point support the idea that our model-based synthesis of KPBS data can be spatially and temporally extrapolated to model ANPP and fuel loads for the Flint Hills ecoregion—the topic of the final Results section.

However, before discussing that, we also need to assess the accuracy of modeled effects of grazing on grassland biomass and fuel loads, a precondition for Flint Hills applications.

#### Modeled effects of grazing on grassland biomass and fuel loads

As Fig. 12 indicated, VELMA also modeled effects of grassland grazing by KPBS resident bison in the southern portion of Kings Creek watershed (code “N” in Fig. 8), where bison grazing encompassed burned and unburned upland and lowland treatments, beginning with the 1991 reintroduction of bison to KPBS tallgrass prairie. Although grazed treatments are not included in the Fig. 11 ANPP analysis, we needed to test the accuracy of VELMA’s grazer model (Fig. 6) because grazing has a major impact on surface fuels and nitrogen cycling.

To that end, we modeled and GIS-sampled upland and lowland standing plant biomass across all subwatersheds within the bison code “N” Kings Creek watershed zone. At the watershed-scale, bison graze year-round at KPBS and are stocked at a rate designed to remove 25% of average ANPP. The annual stocking rate was 1.7 ha/animal unit (au), which is slightly heavier than the 2.1 ha/au that is considered standard for moderate season-long steer grazing in Flint Hills rangeland. That is not an appreciable difference for modeling percent biomass removed at mid-season for an early intensive stocking scheme, provided the stocking rates are similar. Averaged across all years, the simulated KPBS bison herd reduced upland and lowland peak season aboveground plant biomass by 18 and 23%, respectively, compared to ungrazed “K” zone treatments (Fig. 8).

We also tested VELMA’s grazer model against a separate USDA-funded study conducted in small, replicated pastures at KPBS where bison and cattle grazed separately but at equivalent densities (Towne et al. 2005). That study examined grazing differences for cattle and bison when stocked at the same rate and managed the same way for season-long (May–October) grazing. That approach does not reflect the way bison and cattle are managed at the KPBS watershed-scale, nor does it reflect prevalent early intensive stocking schemes in the Flint Hills. Nonetheless, when VELMA’s grazer model was set up using Towne et al. (2005) grazing parameters, VELMA simulated how bison and cattle consumed the equivalent of 52% and 46%, respectively, of upland and lowland peak season plant biomass for ungrazed “K” zone treatments (Fig. 8). Towne et al. (2005) reported similar reductions in potential peak-season grassland biomass for bison (54%) and cattle (46%) during their 10-year KPBS USDA grazing experiment.

VELMA’s flexibility for accurately representing distinctly different tallgrass prairie grazer management options (Ratajczak et al. 2022 versus Towne et al. 2005) bodes well for VELMA’s spatial and temporal extrapolation for modeling Flint Hills cattle grazing effects on grassland plant biomass and fuel loads. Moreover, VELMA’s grazer nitrogen model also ensures that grazer effects are well-integrated with the ecosystem N cycle. This includes partitioning of the grazed fraction of standing live plant biomass N to grazer weight gain, manure, urine, and volatilized N pools (Fig. 6).

### Extrapolation of VELMA KPBS synthesis to Flint hills ecoregion

The results described above demonstrate that VELMA provides an imperfect but serviceably accurate synthesis of KPBS experimental data, thereby establishing a tool for simulating the effects of climate, fire, grazing, topography, and availabilities of soil moisture and N on tallgrass prairie productivity and fuel load dynamics.

In this section, we focus on the use of VELMA to extrapolate its KPBS data synthesis to support Flint Hills prescribed burning smoke dispersion and trajectory modeling for simulating downwind air quality impacts on rural and urban communities, potentially in real-time, and more broadly to assist rural and urban stakeholders and decision makers in their shared goals to better understand, estimate, and balance ecological and air quality trade-offs for actual and hypothetical rangeland burning scenarios.

But before jumping to the ecoregion scale, Fig. 13 is an informative mesoscale performance test of modeled daily fuel load and grassland biomass dynamics during 1993 for the 11.4 km^2^ KPBS Kings Creek watershed (this figure was briefly discussed in a previous section in the context of variable N vs water constraints on ANPP). As the Fig. 13 caption notes, this graph summarizes modeled 1993 lowland dead fuel loads and aboveground live grassland biomass dynamics, averaged across the watershed’s lowland habitats. Those lowland fuel load and biomass dynamics include initially slow decomposition of surface fuels in winter and early spring (a function of temperature and soil moisture constraints), followed by April 9th and May 12th prescribed fires that stimulated rapid grassland biomass growth. Decomposition of surface fuels increased in summer, releasing enough plant-available N to sustain relatively slower growth until peak ANPP was reached in mid-September. At that point live plant biomass began to senesce, feeding a gradual fuel load recovery through late December.

This same virtual tallgrass prairie live and dead biomass dynamic plays out annually across all scales: grid cells to landowner parcels to watershed to ecoregion. The dynamics differ depending on management and climate. Figure 13 is itself an aggregate average of all lowland tallgrass prairie treatments—grazed, ungrazed, burned, and unburned—within the Kings Creek watershed during 1993.

Together, Fig. 9 through Fig. 13 have provided the conceptual and scientific foundations for our extrapolation of VELMA for modeling Flint Hills tallgrass prairie plant biomass and fuel loads dynamics.

Scaling up, Fig. 14 shows VELMA modeled Flint Hills ecoregion fuel load maps (g C/m^2^) for December 31 of 2012, 2016, 2022. These 3 years were chosen to illustrate interannual fuel load variability from among maps developed for 2000 to 2022. Recall that the Flint Hills VELMA grid cell scale is 125 m, requiring over 1.6 million cells to be modeled per simulation year across the 25,730 km^2^ ecoregion.

Takeaways for Fig. 14 are that Flint Hills fuel loads can be highly variable spatially and temporally—e.g., by up to 3 × across years and locations—and that scientifically grounded, spatially explicit fuel load maps will be necessary for accurate Flint Hills air quality modeling assessments.

Figure 15 is one such air quality demonstration for which VELMA fuel load estimates were developed, as described, and applied as inputs to the BlueSky smoke dispersion and trajectory modeling framework (per Larkin et al. 2009) to simulate downwind trajectories of particulate matter and other atmospheric pollutants (e.g., VOCs, NOx, ozone). Shown is a single frame of a BlueSky framework animation in the north central Flint Hills for extant weather conditions and MODIS-detected fires scars on 4/10/2003 at 2:04 pm showing downwind temporal plume development and chemistry (Barnhart et al. 2015).

In summary, the coupling of VELMA fuel load estimates with BlueSky prescribed fire simulations can potentially assist rangeland managers, air quality regulators, and diverse stakeholders in visualizing and understanding options for identifying best management practices for optimizing air quality, tallgrass prairie sustainability, and associated economic benefits for the Flint Hills ecoregion and surrounding downwind communities.

These broader goals are consistent with the multi-stakeholder developed State of Kansas Smoke Management Plan. (State of Kansas 2010). With the potential for routine coupling of ecological and air quality modeling technologies now at hand, those broader stakeholder goals seem quite attainable.

The VELMA-BlueSky modeling work reported here is one output of many long-time collaborative scientific and environmental protection outputs of coauthor partners with EPA-ORD, EPA Region 7, Kansas Department of Health and Environment, Kansas State University, and Konza Prairie Biological Station.

## Conclusions

The process-based VELMA ecohydrological model has previously proven useful for simulating effects of carbon, nutrient, and water cycling on plant-soil–water dynamics across a wide range of ecosystem types, cited herein. When calibrated for the data-rich KPBS, VELMA allowed an analysis of a self-consistent representation of tallgrass prairie ecosystem plant-soil–water interactions and responses to climate and management.

Through its synthesis of diverse KPBS data sets, VELMA extended those data by allowing behaviors of difficult to measure ecosystem components—soil moisture, streamflow, NPP, and fuel loads, for example—to be inferred and mapped across a wide range of spatial and temporal scales: days to decades, and watersheds to ecoregion. Inferred ecosystem components were constrained through the model’s calibration for measured components of the intensively studied KPBS ecosystem. In so doing, VELMA has provided a serviceably accurate portrayal of KPBS experimental treatment responses supporting integrated, process-based assessments of ecosystem responses to change than would be possible using observed data alone. Importantly, these capabilities supported extrapolation of the experimental data in space and time to the Flint Hills tallgrass prairie ecoregion.

Key performance tests of this virtual tallgrass prairie ecosystem included simulations of long-term observed daily streamflow; inferred flow paths and soil moisture levels; interannual variations of observed tallgrass prairie aboveground net primary production (ANPP) coupled with simulated grazer impacts on fuel load dynamics. The generally close correspondence of modeled and observed interannual variations in tallgrass prairie ANPP across upland and lowland burned and unburned landscape-scale treatments revealed VELMA’s internal consistency for modeling integrated N–C-water prairie ecohydrological processes.

For example, ANPP was modeled partly as a function of soil NH_4_ and NO_3_ uptake. But those soil N pools are influenced by simulated rates of soil organic matter decomposition and N mineralization (+ for NH_4_); nitrification of NH_4_ (+ for NO_3_); denitrification (− for NO_3_); transport to streams (− for NH_4_ and NO_3_); and N volatilization during prescribed fires (− for NH_4_ and NO_3_). VELMA simulates interactions among all of these N cycling processes, which are in turn linked with carbon cycling processes, and mediated by plot, hillslope, and watershed-scale hydrological processes. These are complex interactions, dynamically responsive to climate and management, but constrained through system-level ecohydrological feedbacks represented in VELMA. When properly calibrated, those feedbacks enable long-term simulations to proceed in dynamic balance, without unrealistic or fatal model outcomes. Links in the Supplementary Information section provide information on KPBS VELMA model parameter values, simulation drivers, equations, and calibration instructions.

This model-based synthesis and extrapolation of experimental data has significant potential practical applications in Flint Hills environmental decision making. Examples include regional fuel load maps to help rangeland managers factor in downwind air quality impacts of prescribed burning decisions via KDHE’s www.ksfire.org website; and more broadly to assist rural and urban Flint Hills stakeholders and decision makers in their shared goals to better understand, estimate, and balance ecological and air quality trade-offs for actual and hypothetical rangeland burning scenarios.

Our multi-institution team is confident that VELMA Flint Hills fuel load maps, coupled with stakeholder friendly BlueSky air quality modeling support via www.ksfire.org, can help decision makers identify tallgrass prairie management practices that better balance rangeland burning ecological and economic necessities against potential air quality and human health impacts. A key, yet unrealized, goal for this toolset is a multi-partner exploration of how much, where, and when rangeland burning can best be conducted to minimize trade-offs and maximize land-management benefits. These goals are consistent with the multi-stakeholder developed State of Kansas Smoke Management Plan (State of Kansas 2010).

Lending confidence toward these Flint Hills goals are similar VELMA-BlueSky regional applications that compared air quality impacts of recent prescribed fires vs. wildfires for forest ecoregions in Oregon and California (U.S. EPA 2021). At the request of the national Wildland Fire Leadership Council (WFLC) to the U.S. EPA, these multi-model forest fire applications included the use of VELMA to synthesize six decades of ecohydrological data for a high biomass old-growth Douglas-fir forest at the HJ Andrews (HJA) LTER site in western Oregon (https://andrewsforest.oregonstate.edu/). In similar fashion to the KPBS-Flint Hills study, our spatial extrapolation of unadjusted HJA VELMA parameters closely predicted variations in forest ANPP and fuel loads across extreme regional environmental gradients. For example, across a precipitation gradient ranging from 2300 mm/y at the HJA site to 630 mm/y at a much lower biomass ponderosa pine forest site at a 145-km distant site in eastern Oregon (Smithwick et al. 2002) where the U.S. Forest Service routinely applies prescribed fire and fuel reduction treatments to reduce wildfire intensity and air quality issues (VELMA details in section A.7.2.4 in U.S. EPA 2021).

Together, the KPBS-Flint Hills tallgrass prairie and western U.S. forest applications of VELMA-BlueSky underscore the models’ flexibility for providing serviceably accurate insights supportive of environmental decision making across different biomes and regions, and across a range of scales informative to both local and regional resource managers. This final point is important in the context of management needs to address existing uncertainties in the evaluation of alternative actions seeking to balance trade-offs in the protection of ecological resources and human health (State of Kansas 2010; U.S. EPA 2021).

## Figures and Tables

**Table 1:** VELMA driver data sources used for simulating grassland productivity and fuel loads

VELMA Data Type	Source	Year
Daily precipitation (mm) and air temperature (°C)	KPBS for Fig. 10 (Nippert 2023) Flint Hills Daymet 1-km climate grids (Version 4): https://doi.org/10.3334/ORNLDAAC/2129 Flint Hills PRISM 4-km climate grids, Daly et al 2008: https://prism.oregonstate.edu/	1983–1996 1998–2018 2019–2022
Elevation: KPBS 30-m grid; Flint Hills, 125-m grid	USDA Data Gateway DEM: https://datagateway.nrcs.usda.gov/GDGOrder.aspx	All years
Land cover type	US National Land Cover Data set, 2001 https://www.usgs.gov/centers/eros/science/national-land-cover-database	2000–2022
Flint Hill fire-scar data	MODIS burned area products: https://modis-fire.umd.edu/ba.html	2000–2022
Kings Creek Stream Hydrology	Dodds 2023. ASD01 Stream discharge for Kings Creek measured at USGS gaging station Environmental Data Initiative. http://dx.doi.org/10.6073/pasta/70dc45ef3b0dd58ec020fe32c3ab2d82	1983–1996
Aboveground NPP spatial and temporal patterns	Briggs and Knapp 1995; Knapp et al.1998	1983–1996
Groundwater Hydrology	Costigan et al. 2015	1983–1996
Grassland biomass C and N stocks (g/m^2^), annual net primary production (g N/m^2^/y)	Briggs and Knapp 1995; Blair et al. 1998; Knapp et al. 1998	VELMA biomass initializations for KPBS in 1983, and for Flint Hills in 2000
Soil properties: depth and C and N stocks (g/m^2^) by topographic position (upland, midland, lowland)	Knapp et al. 1998; Ransom et al. 1998; Blair et al. 1998	VELMA model soil initializations for KPBS 1983, and for Flint Hills in 2000

## Data Availability

Simulated ecohydrological and air quality data is provided within the manuscript and in supplementary information files located here: https://sciencehub.epa.gov/sciencehub/datasets/4398. All simulated model data are based on published sources and publicly available forcing variables cited in the manuscript.

## References

[R1] Abdelnour A, Stieglitz M, Pan F, McKane R (2011) Catchment hydrological responses to forest harvest amount and spatial pattern. Water Resour Res 47:W09521

[R2] Abdelnour A, McKane RB, Stieglitz M, Pan F, Cheng Y (2013) Effects of harvest on carbon and N dynamics in a Pacific Northwest forest catchment. Water Resour Res 49:1292–1313.

[R3] Allen MS, Palmer MW (2011) Fire history of a prairie/forest boundary: more than 250 years of frequent fire in a North American tallgrass prairie. J Veg Sci 22:436–444

[R4] Barnhart B, Pettus P, Halama J, McKane R, Mayer P, Djang K, Brookes A, Moskal LM (2021) Modeling the hydrologic effects of watershed-scale green roof implementation in the Pacific Northwest United States. J Environ Manage 277:111418 10.1016/j.jenvman.2020.111418 PMC8237799 33080432

[R5] Barnhart BL, McKane R, Brookes A, Schumaker N, Papenfus M, Pettus P, Halama J, Powers B, Djang K, Groskinsky B, Grier G, Hawkins A, Tapp J, Watson D, Gross T, Goodin D, Mohler R (2015) Integrated modeling to assess the ecological and air quality trade-offs of agricultural burning in the Flint Hills of eastern Kansas. Abstract, American Geophysical Union Fall Meeting, December 14–18, 2015, San Francisco, CA. https://ui.adsabs.harvard.edu/abs/2015AGUFMIN11B1780B

[R6] Blair JM (1997) Fire, N availability, and plant response in grasslands: a test of the transient maxima hypothesis. Ecology 78:2359–2368

[R7] Blair JM, Seastedt TR, Rice CW, Ramundo R (1998) Terrestrial nutrient cycling in tallgrass prairie. Grassland dynamics: long-term ecological research in tallgrass prairie. Oxford University Press, New York, pp 222–243

[R8] Briggs JM, Knapp AK (1995) Interannual variability in primary production in tallgrass prairie: climate, soil moisture, topographic position, and fire as determinants of aboveground biomass. Am J Bot 82:1024–1030

[R9] Byun D, Schere KL (2006) Review of the governing equations, computational algorithms, and other components of the Models-3 community multiscale air quality (CMAQ) modeling system. Appl Mech Rev 59:51–77

[R10] Cimorelli AJ, Perry SG, Venkatram A, Weil JC, Paine RJ, Wilson RB, Lee RF, Peters WD, Brode RW (2005) AERMOD: a dispersion model for industrial source applications. Part I: general model formulation and boundary layer characterization. J Appl Meteorol Climatol 44(5):682–693

[R11] Collins SL, Nippert JB, Blair JM, Briggs JM, Blackmore P, Ratajczak Z (2021) Fire frequency, state change and hysteresis in tallgrass prairie. Ecol Lett 24:636–647 33443318 10.1111/ele.13676

[R12] Costigan KH, Daniels MD, Dodds WK (2015) Fundamental spatial and temporal disconnections in the hydrology of an intermittent prairie headwater network. J Hydrol 522:305–316

[R13] Cuddington K, Fortin MJ, Gerber LR, Hastings A, Liebhold A, O’connor M, Ray C (2013) Process-based models are required to manage ecological systems in a changing world. Ecosphere 4:1–12

[R14] Daly C, Halbleib M, Smith JI, Gibson WP, Doggett MK, Taylor GH, Curtis J, Pasteris PP (2008) Physiographically sensitive mapping of climatological temperature and precipitation across the conterminous United States. Int J Climatology 28:2031–2064

[R15] Konza Prairie Data Catalog: AWE01 Meteorological data from the Konza Prairie headquarters weather station. http://lter.konza.ksu.edu/content/awe01-meteorological-data-konza-prairie-headquarters-weather-station

[R16] Dodds WK 2023. ASD01 Stream discharge for Kings Creek measured at USGS gaging station Environmental Data Initiative. 10.6073/pasta/70dc45ef3b0dd58ec020fe32c3ab2d82

[R17] Duncan ZM, Tajchman AJ, Ramirez MP, Lemmon J, Hollenbeck WR, Blasi DA, Fick WH, Olson KC (2021) Effects of prescribed fire timing on grazing performance of yearling beef cattle, forage biomass accumulation, and plant community characteristics on native tallgrass prairie in the Kansas Flint Hills. Transl Animal Sci 5:txab077 10.1093/tas/txab077 PMC8494120 34632310

[R18] Dunne T, Leopold LB (1978) Water in environmental planning. Macmillan

[R19] Eisele L, Schimel DS, Kapustka LA, Parton WJ (1989) Effects of available P and N: P ratios on non-symbiotic dinitrogen fixation in tallgrass prairie soils. Oecologia 79:471–474 28313480 10.1007/BF00378663

[R20] Freeman CC, Hulbert LC (1985) An annotated list of the vascular flora of Konza Prairie research natural area, Kansas. Trans Kans Acad Sci 1903:84–115

[R21] Gelbard JL (2003) Grasslands at a Crossroads: Protecting and Enhancing Resilience to Climate Change. In: Hansen L, Biringer JL, Hoffman JR (eds) Buying time: a user’s manual for building resistance and resilience to climate change in natural systems. World Wildlife Fund, Berlin

[R22] Halama J, McKane R, Barnhart B, Pettus P, Brookes A, Djang K, Phan V, Chokshi S, Graham J (2023) Improved urban runoff prediction using high-resolution land-use, imperviousness, and stormwater infrastructure data applied to a process-based ecohydrological model. PLOS Water 2(11):e0000155 10.1371/journal.pwat.0000155 PMC11110540 38783969

[R23] Halama JJ, McKane RB, Barnhart BL, Pettus PP, Brookes AF, Adams AK, Gockel CK, Djang KS, Phan V, Chokshi SM, Graham JJ (2024) Watershed analysis of urban stormwater contaminant 6PPD-Quinone hotspots and stream concentrations using a process-based ecohydrological model. Front Environ Sci 12:1364673 10.3389/fenvs.2024.1364673 PMC11151736 38845698

[R24] Hobbs NT, Schimel DS, Owensby CE, Ojima DS (1991) Fire and grazing in the tallgrass prairie: contingent effects on nitrogen budgets. Ecology 72(4):1374–1382

[R25] Hoghooghi N, Golden HE, Bledsoe BP, Barnhart BL, Brookes AF, Djang KS, Halama JJ, McKane RB, Nietch CT, Pettus PP (2018) Cumulative effects of low impact development on watershed hydrology in a mixed land-cover system. Water 10:991 31396407 10.3390/w10080991 PMC6687309

[R26] Homer C, Huang C, Yang L, Wylie B, Coan M (2004) Development of a 2001 national land-cover database for the United States. Photogramm Eng Remote Sens 70:829–840

[R27] Hulbert LC (1988) Causes of fire effects in tallgrass prairie. Ecology 69(1):46–58

[R28] Jackson RB, Canadell J, Ehleringer JR, Mooney HA, Sala OE, Schulze ED (1996) A global analysis of root distributions for terrestrial biomes. Oecologia 108:389–411 28307854 10.1007/BF00333714

[R29] Knapp AK, Briggs JM, Blair JM, Turner CL (1998) Patterns and controls of aboveground net primary production in tallgrass prairie. Grassland dynamics: long-term ecological research in tallgrass prairie. Oxford University Press, New York

[R30] Knapp AK, Blair JM, Briggs JM, Collins SL, Hartnett DC, Johnson LC, Towne EG (1999) The keystone role of bison in North American tallgrass prairie: Bison increase habitat heterogeneity and alter a broad array of plant, community, and ecosystem processes. Bioscience 49:39–50

[R31] Kollmorgen WM, Simonett DS (1965) Grazing operations in the Flint Hills–bluestem pastures of Chase County, Kansas. Ann Assoc Am Geogr 55(2):260–290

[R32] Konza Prairie LTER Data Explorer, Data set ID:4, EML revision ID:19, Data sources: APT011.csv APT01 Daily precipitation amounts measured at multiple sites across Konza prairie. http://lter.konza.ksu.edu/content/apt01-daily-precipitation-amounts-measured-multiple-sites-across-konza-prairie

[R33] Kuzyakov Y, Xu X (2013) Competition between roots and microorganisms for nitrogen: mechanisms and ecological relevance. New Phytol 198(3):656–669 23521345 10.1111/nph.12235

[R34] Larkin NK, O’Neill SM, Solomon R, Raffuse S, Strand T, Sullivan DC, Krull C, Rorig M, Peterson J, Ferguson SA (2009) The BlueSky smoke modeling framework. Int J Wildland Fire 18(8):906–920

[R35] Mast MA, Turk JT (1999) Environmental characteristics and water quality of hydrologic benchmark network stations in the West-Central United States, 196395: U.S. Geological Survey Circular 1173–C, p. 105

[R36] McKane RB, Rastetter EB, Shaver GR, Nadelhoffer KJ, Giblin AE, Laundre JA, Chapin FS III (1997) Climatic effects on tundra carbon storage inferred from experimental data and a model. Ecology 78(4):1170–1187

[R37] McKane RB, Brookes AF, Djang KS, Halama JJ, Pettus PB, Barnhart BL, Russell MJ, Vache KB, Bolte JB (2020) An integrated multi-model decision support framework for evaluating ecosystem-based management options for coupled human-natural systems. Ecosystem-based management, ecosystem services and aquatic biodiversity: Theory, tools and applications. Springer, Cham, pp 255–274

[R38] McKane RB, Brookes A, Djang K, Stieglitz M, Abdelnour A, Pan F, Halama JJ, Pettus P, Phillips D (2014) Visualizing ecosystem land management assessments (VELMA) v. 2.0: User manual and technical documentation. Document control number L-PESD-30840-QP-1–2. Corvallis, OR: U.S. Environmental Protection Agency, National health and environmental effects research laboratory. https://www.epa.gov/sites/production/files/2016-01/documents/velma_2.0_user_manual.pdf

[R39] McKane R; Barnhart B, Halama J, Pettus P, Brookes A, Ebersole J, Djang K, Blair G, Hall J, Kane J, Swedeen P, Benson L (2016) Nisqually community forest VELMA modeling. Presentation, 2016 South Sound Science Symposium, September 20, 2016, Olympia, WA.

[R40] McKane RB; Halama J, Pettus P, Barnhart B, Brookes A, Djang K, Blair G, Hall J, Kane J, Swedeen P, Benson L, (2018) How visualizing ecosystem land management assessments (VELMA) modeling quantifies co-benefits and trade-offs in community forest management. Presented at Northwest Community Forest Forum, May 10–11, 2018, Astoria, OR.

[R41] McKane RB, Barnhart BL, Pettus P, Halama JJ, Brookes A, Djang K, Khangaonkar T, Kaplan I, Harvey C, Morzaria Luna H, Schmidt M, Howe E, Levin P, Francis T, Baker J, Stanley S, Hume C. A Science-Governance partnership for integrating ecosystem services into puget sound restoration planning. ACES 2018 Conference, Washington, DC, December 03–06, 2018.

[R42] McKane RB, Brookes AF, Halama JJ, Djang KS, Barnhart BL, Pettus PB, Phan V (2022) VELMA 2.1 Supplement to VELMA 2.0 User Manual. U.S. Environmental Protection Agency, EPA/600/B-22/024.

[R43] McKane RB, Barnhart BB (2017) VELMA training and technical transfer for Flint Hills applications by EPA Region 7 and Kansas Department of Health and Environment, Lenexa, KS, February 2017

[R44] Mohler RL, Goodin DG (2012) Mapping burned areas in the Flint Hills of Kansas and Oklahoma, 2000—2010. Great Plains Research, pp.15–25. https://www.jstor.org/stable/23779865

[R45] Nippert J (2023) AWE01 Meteorological data from the Konza prairie headquarters weather station. Environmental Data Initiative. 10.6073/pasta/743c6b205e38a087bc54925ed258f549

[R46] ODFW (Oregon Department of Fish and Wildlife) (2022) 2022 assessment of naturally produced summer steelhead in the Umpqua River basin. Science Bulletin 2022–1. ODFW, Salem

[R47] Pan F, Stieglitz M, McKane RB (2012) An algorithm for treating flat areas and depressions in digital elevation models using linear interpolation. Water Resources Res, 48(6). 10.1029/2011WR010735

[R48] PRISM Climate Group, Oregon State University, https://prism.oregonstate.edu. Maps created 2019–2022.

[R49] Putnam JE, Lacock DL, Schneider DR, Carlson MD, Dague BJ (1996) Water resources data, Kansas, water year 1995: U.S. Geological Survey Water-Data Report KS-95–1:488.

[R50] Ransom MD, Rice CW, Todd TC, Wehmueller WA (1998) Soils and soil biota. Grassland dynamics: long-term ecological research in tallgrass prairie. Oxford University Press, New York, pp 48–66

[R51] Rastetter EB, Ryan MG, Shaver GR, Melillo JM, Nadelhoffer KJ, Hobbie JE, Aber JD (1991) A general biogeochemical model describing the responses of the C and N cycles in terrestrial ecosystems to changes in CO2, climate, and N deposition. Tree Physiol 9:101–126 14972859 10.1093/treephys/9.1-2.101

[R52] Ratajczak Z, Nippert JB, Briggs JM, Blair JM (2014) Fire dynamics distinguish grasslands, shrublands and woodlands as alternative attractors in the Central Great Plains of North America. J Ecol 102:1374–1385

[R53] Ratajczak Z, Collins SL, Blair JM, Koerner SE, Louthan AM, Smith MD, Taylor JH, Nippert JB (2022) Reintroducing bison results in long running and resilient increases in grassland diversity. Proc Natl Acad Sci 119:e2210433119 10.1073/pnas.2210433119 PMC9457053 36037376

[R54] Rollins MG (2009) LANDFIRE: a nationally consistent vegetation, wildland fire, and fuel assessment. Int J Wildland Fire 18:235–249

[R55] Sacks JD, Lloyd JM, Zhu Y, Anderton J, Jang CJ, Hubbell B, Fann N (2018) The environmental benefits mapping and analysis program-community edition (BenMAP–CE): a tool to estimate the health and economic benefits of reducing air pollution. Environ Model Softw 104:118–129 29962895 PMC6022291

[R56] Seastedt TR, Knapp AK (1993) Consequences of nonequilibrium resource availability across multiple time scales: the transient maxima hypothesis. Am Nat 141:621–633 19426001 10.1086/285494

[R57] Smithwick EA, Harmon ME, Remillard SM, Acker SA Franklin JF (2002) Potential upper bounds of carbon stores in forests of the Pacific Northwest. Ecol App 12(5):1303–1317

[R58] State of Kansas (2010) Flint Hills Smoke Management Plan, December 2010. Link: Flint_Hills_SMP_v10FINAL PDF. www.ksfire.org

[R59] Towne EG, Hartnett DC, Cochran RC (2005) Vegetation trends in tallgrass prairie from bison and cattle grazing. Ecol Appl 15:1550–1559

[R60] U.S. EPA (2021) Comparative Assessment of the Impacts of Prescribed Fire Versus Wildfire (CAIF): A Case Study in the Western U.S. U.S. Environmental Protection Agency, Washington, DC, EPA/600/R-21/044. https://cfpub.epa.gov/si/si_public_record_report.cfm?Lab=CPHEA&dirEntryId=350839&fed_org_id=111

[R61] Yee S, Bousquin J, Bruins R, Canfield TJ, DeWitt TH, de Jesús-Crespo R, Dyson B, Fulford R, Harwell M, Hoffman J, Littles CJ, Johnston JM, McKane RB, Green L, Russel M, Sharpe L, Seeteram N, Tashie A, Williams K (2017) Practical strategies for integrating final ecosystem goods and services into community decision-making. (EPA/600/R-17/266). Washington, DC: U.S. Environmental Protection Agency. https://nepis.epa.gov/Exe/ZyPURL.cgi?Dockey=P100SGRC.txt.

